# Coating silicon catheters with the optimized and stable carotenoid bioproduct from *Micrococcus luteus* inhibited the biofilm formation by multidrug-resistant *Enterococcus faecalis* via downregulation of *GelE* gene expression

**DOI:** 10.1186/s12934-025-02808-8

**Published:** 2025-08-18

**Authors:** Khaled B. Al-Monofy, Ahmed A. Abdelaziz, Amal M. Abo-Kamar, Lamiaa A. Al-Madboly, Mahmoud H. Farghali

**Affiliations:** Department of Microbiology and Immunology, Faculty of Pharmacy, Tanta University, Tanta, Egypt

**Keywords:** *Micrococcus luteus*, Β-carotene, Optimization, One-factor-at-a-time, *Enterococcus faecalis*, Gelatinase

## Abstract

**Background:**

Microbial carotenoids have gained industry interest due to their safety and diverse biological activities; however, the low yield of carotenoids hinders their applications. Hence, this study focused on optimizing carotenoid pigment production from *Micrococcus luteus* strains by studying 54 physical and chemical independent conditions. The chronic infections by *Enterococcus faecalis* are related to its ability to form biofilms on the surface of several implanted medical devices, such as urinary catheters. Therefore, the potential antibacterial and antibiofilm activities of the purified pigment against *E. faecalis* were investigated in our study.

**Results:**

Using one-factor-at-a-time experiments, the top-examined parameters were tryptic soya broth (TSB), agitation, temperature, pH, incubation time, inoculum size (IS), sodium chloride, tryptophan, glycerol, tryptone, glutaric acid, toluene, ferric sulphate, and disodium hydrogen phosphate. The data from the Plackett-Burman design showed that temperature, sodium chloride, tryptophan, and toluene were fundamental factors controlling carotenoid production. The conditions for the upstream process were determined via response surface methodology design, which included TSB medium, agitation speed of 120 rpm, temperature of 32.5 °C, pH = 7, incubation time of 96 h, 2% IS, sodium chloride (12.5 g/l), tryptophan (12.5 g/l), glutaric acid (5 g/l), toluene (12.5%), and disodium hydrogen phosphate (5 g/l). Submerged fermentation model validation using the M6 isolate (accession number of PP197163) revealed an increase in carotenoid production up to 6-fold (1.2 g/l). The produced pigment was purified and characterized as β-carotene, and the stability study showed that the extracted β-carotene was stable for a year in dimethyl sulfoxide at 4 °C. The MTT test data proved that the pigment was safe on human dermal fibroblasts with an IC_50_ equal to 542.7 µg/ml. For the first time, it was reported that the stable purified β-carotene exhibited powerful antibacterial activity against multidrug-resistant (MDR) *E. faecalis*, with inhibition zones ranging from 13 to 32 mm and minimum inhibitory concentrations (MICs) ranging from 3.75 to 30 µg/ml at safe concentrations. In addition, it was found that our stable purified β-carotene showed up to 94% inhibition in biofilm formation by strong biofilm-forming *E. faecalis*. In addition, the β-carotene-coated catheter manifested a lower biofilm formation by *E. faecalis* by up to 75.3%. Moreover, crystal violet staining, dual staining, and fluorescence staining techniques displayed immature biofilms of *E. faecalis* when treated with 0.25 and 0.5 MICs of β-carotene. The mechanistic pathway for the purified β-carotene’s antibiofilm activity was strongly linked to the inhibition of gelatinase enzyme production (up to 100% inhibition) as manifested phenotypically, genotypically, and by molecular docking.

**Conclusion:**

This work provided a deeper insight into optimizing carotenoid production from *M. luteus* by investigating the influence of 54 diverse conditions. Also, this is the first time to report the antibacterial and antibiofilm actions of the stable purified microbial β-carotene against strong biofilm-forming MDR *E. faecalis* colonizing urinary catheters.

**Supplementary Information:**

The online version contains supplementary material available at 10.1186/s12934-025-02808-8.

## Background

As secondary metabolites, pigments are created by diverse microorganisms and serve a variety of purposes, such as protecting against ultraviolet radiation, supporting signaling that regulates gene expression, facilitating iron absorption, and aiding in photosynthesis [1]. Many microbes, such as bacteria, algae, yeasts, and fungi, are known to yield natural pigments. These microbes have been shown to synthesize pigments belonging to various categories, such as carotenoids, flavones, phenazines, violaceins, melanins, and others [2].

Recently, the industry has become interested in using natural pigments for human use due to their numerous benefits, including biodegradability and lack of harmfulness, in comparison with synthetic ones [3]. The quick extraction and easy scaling up of microbial pigments make them the most favored natural pigments [4]. Additionally, these pigments are now an essential part of our daily life and have several applications in a series of industries, such as agriculture, cosmetics, and food [5].

Carotenoids are among the significant and often used pigments derived from microbial and plant sources, as around 1100 carotenoids from various sources are known [6]. They are isoprenoid polyene compounds, consisting of 40 carbon atoms and 2 terminal rings, which generate lipid-soluble yellow, orange, and red pigments [7]. Two types of carotenoids exist: oxygen-containing xanthophylls, including zeaxanthin, lutein, and astaxanthin, and hydrocarbon carotenes, which include lycopene, α-carotene, and β-carotene [7]. Recent reports found that carotenoids have anticancer, antibacterial, and antioxidant activities [8].

Among carotenogenic bacteria, *Micrococcus luteus* has gained notice for producing many strain-dependent carotenoid pigments. *M. luteus* produces several types of carotenoids, including zeaxanthin, sarcinaxanthin, neoxanthin, lycopene, β-carotene, lycoxanthin, lycopene epoxide, tetra-dehydrocarotenoid, antheraxanthin diester, and lycophyll diester [9, 10]. Based on the available data, environmental stress conditions can affect the biosynthesis of carotenoids and create variations in their profiles and quantities. Hence, in order to get strategies that motivate microorganisms to adjust carotenoid synthesis, it is crucial to manipulate the cultivation parameters [11]. In general, stressful environmental conditions provoke the synthesis of carotenoid pigments as a self-protective mechanism against the existing stress [12], and according to Kot et al. (2018), variations in salinity, pH, and temperature trigger carotenogenesis in some bacterial species [13]. Therefore, this study focused on optimizing carotenoid pigment production from *M. luteus* to explore its potential activities.

Gram-positive *Enterococcus faecalis* is a commensal bacterium that is present in humans, animals, and a variety of settings. However, it can also function as an opportunistic pathogen, leading to a number of diseases, such as infective endocarditis and catheter-associated urinary tract infections. It is a major cause of nosocomial infections globally and accounts for 80–90% of enterococcal infections [14]. The emergence of multidrug-resistant (MDR) *E. faecalis* strains, which are resistant to the majority of antimicrobial drugs, has made hospital-acquired infections a global concern, necessitating innovative, focused treatments [15]. According to Veerachamy et al. (2014), *E. faecalis* frequently forms biofilms on the outer layers of several implanted medical devices that cause chronic infections [15]. These biofilms play a significant pathogenicity factor since these bacteria can persist in the human body for an extended period and are resistant to antibiotic treatment and immune system defence [14]. Consequently, the current study examined the ability of the produced pigment from *M. luteus* as a dual antibacterial and antibiofilm agent against strong biofilm-forming MDR *E. faecalis.*

## Materials and methods

### Isolation of *M. luteus* and screening for carotenoid pigment production

Thirty-three different agricultural soil samples, taken from Zifta City, Egypt, were suspended with normal saline and allowed to settle (1 gm of soil + 9 ml diluent). Then, the upper phase was serially diluted (dilution factor = 10) with normal saline in sterile test tubes (*n* = 5), and a volume of 1 ml of the diluent was taken from the last test tube and inoculated into a sterile petri dish. After that molten nutrient agar (19 ml) was poured and mixed with the diluted sample, and then cultured for 48 h at 37 °C. After the isolation of yellow-pigmented colonies, they were identified using the Gram staining technique and a series of biochemical tests. The confirmation of the identity of the isolated colonies was accomplished by conducting matrix-assisted laser desorption ionization-time of flight mass spectroscopy (MALDI-TOF MS) on representative isolates (M6, M9, and M10) [16, 17]. The isolated *M. luteus* strains were investigated for carotenoid production by incubating 1% inoculum of bacteria (0.5 McFarland) into Falcon tubes (50 ml) in a rotary shaker for 24 h at 37 °C, using nutrient broth (NB) as a growth medium (final volume = 25 ml). After that, the media were centrifuged at 10.000 *g* for 30 min at 4 °C, and the carotenoid pigment was extracted from pellets by adding 2 ml of methanol. Finally, the optical density (OD) at 450 nm was measured for each methanolic extract [18].

### Molecular identification of M6 isolate

The most pigment-producing strain (M6 isolate) was molecularly characterized using the standardized method of Kathiresan et al. (2014) [19]. In brief, using the GeneJet Genomic DNA (deoxyribonucleic acid) Purification Mini Kit (Thermo Fisher Scientific) and following the manufacturer’s instructions, the whole DNA from the broth culture of the tested strain was extracted and purified. Afterward, a 0.2 ml optical-grade polymerase chain reaction (PCR) tube was used to carry out a *16 S rRNA* amplification reaction utilizing 16 S primers: forward primer 20 F (5′-AGAGTTTGATCMTGGCTCAG-3′) and reverse primer 1492R (5′-GGTTACCTTGTTACGACTT-3′). The amplification was carried out using a final volume of 25 µl of PCR reaction mixture containing 0.3 µl of each primer (10 pmol/µl), 1 µl of DNA template (50–100 ng), 10.9 µl of distilled water, and 12.5 µl of master mix (Sinaclon Cat. No. MM2062). The PCR was conducted in a thermal cycler (Creacon Technologies, Netherlands). The steps of PCR include an initial denaturation step at 95 °C for 5 min, followed by 30 cycles of denaturation at 95 °C for 30 s, an annealing step at 52 °C for 45 s, and an extension step at 72 °C for 90 s, and finally a final extension step at 72 °C for 10 min. The PCR product was allowed to run on 1% agarose in TAE buffer [40 mmol Tris, 20 mmol acetic acid, 1 mmol EDTA (pH = 8)], stained with ethidium bromide, and visualized using a gel documentation system (GelDoc-It, UVP, England). Using the QIAquick^®^ PCR Purification Kit, the PCR product was purified for sequencing. Utilizing Molecular Evolutionary Genetics Analysis (MEGA X) software, the *16 S rRNA* gene’s nucleotide sequence was processed. To calculate similarity percentages, the *16 S rRNA* gene sequence was retrieved using the Basic Local Alignment Search Tool (BLASTn) and compared to previously published sequences of bacteria in the National Center for Biotechnology Information (NCBI) databases. The Molecular Evolutionary Genetics Analysis (MEGA X) software’s CLUSTALW algorithm was used to achieve multiple DNA sequence alignments of a chosen *16 S rRNA*, and the phylogenetic tree was formed based on the maximum-likelihood method [20].

### One-factor-at-a-time (OFAT) experiments

The influence of different parameters on the yield of the carotenoid pigment was explored using the OFAT technique. For each physicochemical parameter considered (Table S1), a Falcon tube (50 ml) containing 25 ml of medium and the inoculum volume (0.5 McFarland) was 1% of the total volume used for the optimization procedures. Also, the incubation lasted for 24 h at 37 °C with an agitation speed of 120 rpm. Firstly, different media were screened for maximum carotenoid pigment production, and the best medium was selected for the subsequent parameters [21]. Assessment of the effect of the parameter was carried out by pigment extraction from cell pellets after the centrifugation step and measuring OD at 450 nm [22].

### The Plackett–Burman design (PBD)

The parameters of agitation, temperature, pH, incubation time, inoculum size (IS), sodium chloride, tryptophan, glycerol, tryptone, glutaric acid, toluene, ferric sulphate, and disodium hydrogen phosphate were examined using PBD for evaluating the main factors controlling the production of carotenoid by the M6 isolate. The design was performed using Minitab software, and each parameter had two levels [21]. The obtained regression equation was:

$$\begin{aligned} {\text{Actual}} & ={\text{ }} - 0.{\text{115 }}+~0.000{\text{342}}~{\text{Agitation}}~\left( {{\text{rpm}}} \right){\text{ }}+~0.0{\text{1158}}~{\text{Temperature}}~\left( {^\circ {\text{C}}} \right){\text{ }}+~0.0{\text{145}}~{\text{pH}} \\ & \quad +~0.000{\text{236}}~{\text{Incubation}}~{\text{time}}~\left( {\text{h}} \right){\text{ }}+~0.0{\text{41}}0~{\text{IS}}~\left( \% \right){\text{ }}+~0.0{\text{27}}00~{\text{Sodium}}~{\text{chloride}}~\left( {{\text{g}}/{\text{l}}} \right) \\ & \quad +~0.0{\text{27}}00~{\text{Tryptophan}}~\left( {{\text{g}}/{\text{l}}} \right){\text{ }}+~0.00{\text{42}}0~{\text{Glycerol}}~\left( \% \right){\text{ }}+~0.00{\text{62}}0~{\text{Tryptone}}~\left( {{\text{g}}/{\text{l}}} \right) \\ & \quad +~0.00{\text{38}}0~{\text{Glutaric}}~{\text{acid}}~\left( {{\text{g}}/{\text{l}}} \right){\text{ }}+~0.0{\text{29}}00~{\text{Toluene}}~\left( \% \right){\text{ }}+~0.0{\text{1}}0{\text{6}}0~{\text{Ferric}}~{\text{sulphate}}~\left( {{\text{g}}/{\text{l}}} \right) \\ & \quad +~0.00{\text{62}}0~{\text{Disodium}}~{\text{hydrogen}}~{\text{phosphate}}\left( {{\text{g}}/{\text{l}}} \right) \\ \end{aligned}$$

### Response surface methodology (RSM) design

The interaction between code factors (temperature, sodium chloride, tryptophan, and toluene) was evaluated by Central Composite Design (CCD) using Minitab software for determining the best conditions for the production of carotenoid by M6 isolate, where each factor had 5 levels [21]. The contour plot was used to show how the components interacted, and the obtained regression equation was:

$$\begin{aligned} {\text{Actual}} & =~~ - {\text{6}}.{\text{866 }}+~0.{\text{38816}}~{\text{Temperature}}~\left( {^\circ {\text{C}}} \right){\text{ }}+~0.{\text{111}}0{\text{5}}~{\text{Sodium}}~{\text{chloride}}~\left( {{\text{g}}/{\text{l}}} \right) \\ & \quad +~0.0{\text{676}}0~{\text{Tryptophan}}~\left( {{\text{g}}/{\text{l}}} \right){\text{ }}+~0.0{\text{9527}}~{\text{Toluene}}~\left( \% \right) \\ & \quad - ~0.00{\text{5798}}~{\text{Temperature}}~\left( {^\circ {\text{C}}} \right)*{\text{Temperature}}~\left( {^\circ {\text{C}}} \right) \\ & \quad - ~0.00{\text{3691}}~{\text{Sodium}}~{\text{chloride}}~\left( {{\text{g}}/{\text{l}}} \right)*{\text{Sodium}}~{\text{chloride}}~\left( {{\text{g}}/{\text{l}}} \right) \\ & \quad - ~0.00{\text{2192}}~{\text{Tryptophan}}~\left( {{\text{g}}/{\text{l}}} \right)*{\text{Tryptophan}}~\left( {{\text{g}}/{\text{l}}} \right) \\& \quad - ~0.00{\text{3348}}~{\text{Toluene}}~\left( \% \right)*{\text{Toluene}}~\left( \% \right) \\ & \quad - 0.000{\text{444}}~{\text{Temperature}}~\left( {^\circ {\text{C}}} \right)*{\text{Sodium}}~{\text{chloride}}~\left( {{\text{g}}/{\text{l}}} \right) \\ & \quad +~0.000000~{\text{Temperature}}~\left( {^\circ {\text{C}}} \right)*{\text{Tryptophan}}~\left( {{\text{g}}/{\text{l}}} \right)\\ & \quad - ~0.000{\text{2}}00~{\text{Temperature}}~\left( {^\circ {\text{C}}} \right)*{\text{Toluene}}~\left( \% \right) \\ & \quad - ~0.0000{\text{67}}~{\text{Sodium}}~{\text{chloride}}~\left( {{\text{g}}/{\text{l}}} \right)*{\text{Tryptophan}}~\left( {{\text{g}}/{\text{l}}} \right) \\ & \quad +~0.000{\text{133}}~{\text{Sodium}}~{\text{chloride}}~\left( {{\text{g}}/{\text{l}}} \right)*{\text{Toluene}}~\left( \% \right)\\ & \quad - ~0.0000{\text{44}}~{\text{Tryptophan}}~\left( {{\text{g}}/{\text{l}}} \right)*{\text{Toluene}}~\left( \% \right) \\ \end{aligned}$$

### Submerged fermentation model validation and extraction of carotenoid

The validity of the optimization was carried out by incubating the M6 isolate in optimized conditions (TSB) medium, agitation speed of 120 rpm, temperature of 32.5 °C, pH = 7, incubation time of 96 h, 2% IS, sodium chloride (12.5 g/l), tryptophan (12.5 g/l), glutaric acid (5 g/l), toluene (12.5%), and disodium hydrogen phosphate (5 g/l) and unoptimized conditions (NB medium, temperature of 25 °C, pH = 5, incubation period of 24 h, and 1% IS) conditions with a final volume of 250 ml. After incubation, the bacterial culture was centrifuged (10.000 g) for 30 min, and pellets were gathered and combined with methanol (4:1 solvent/pellets, v/w). After vortexing (1 min), this mixture was placed in a water bath for 15 min (60 °C). The resultant supernatant was centrifuged (10.000 g) for 15 min at 4 °C, and then the remaining pellets were mixed again with methanol until the pellets lost their yellow color. Finally, the methanolic extracts from optimized and unoptimized conditions were evaporated in a porcelain dish at 70 °C, weighed, and compared to each other [18, 21].

### Purification and characterization of the extracted carotenoid

#### Silica gel column chromatography

The produced carotenoid pigment was purified by silica gel column chromatography (60–120 mesh size). At first, the pigment was eluted with n-hexane, then acetone was added to elevate the polarity of the solvent system, and the yellow fractions were collected together from the column. Finally, the purified pigment was evaporated in a porcelain dish at 70 °C, weighed, and then stored at 4 °C for subsequent examinations [23].

#### Thin-layer chromatography (TLC)

The selection of the proper solvent for TLC is essential and depends on a degree of trial and error. After using a mixture of n-hexane: acetone solution with a ratio of 6:4 (v/v) as a solvent system, the purified pigment was dotted onto TLC plates (silica gel 60 matrix with aluminum support, thickness: 200 μm, particle size: 10–12 μm, pore size: 60 Å medium pore diameter), and the retention factor (R_f_) value was measured [24].

#### Differential scanning calorimetry (DSC)

The purity of the extracted pigment was investigated using the DSC. A quantity of 5 mg of the purified pigment was placed into a zero alumina pan, sealed, and then put into the differential scanning calorimeter (Discovery DSC 25-TA, USA). The thermal analysis was performed between 0 and 250 °C (heating rate = 10 °C/min), and the resulting melting curve was processed with TRIOS software [25].

#### Fourier-transform infrared spectroscopy (FTIR)

The functional chemical groups existing in the purified pigment were determined through the spectrum of FTIR, which was compared to the obtained spectrum of the standard β-carotene (Sigma-Aldrich). In brief, to create a homogenous pellet, 100 mg of potassium bromide and 1 mg of the dried carotenoid pigment were combined, and analyzed by means of an FTIR spectrometer (FTIR SPECTROMETER 4100 JASCO-JAPAN) with a resolution of 4 cm^−1^ from 4000 to 400 cm^−1^ [26].

#### High-performance liquid chromatography (HPLC)

The potential β-carotene identity of the produced pigment was further emphasized by HPLC. An extract volume of 20 µl was introduced into a Hypersil ODS column (C18, 250 × 4.6 mm, 5 μm; Thermo Scientific). The column was then eluted with a mixture of acetonitrile and methanol (10:90 v/v) at a temperature of 30 °C and a flow rate of 1.5 ml/min. A diode array detector was utilized for the detection, which was done between 400 and 500 nm, and the result was compared to standard β-carotene [27].

#### Stability study of the purified β-carotene

The stability of the produced β-carotene was checked in dimethyl sulfoxide (DMSO), methanol, and ethanol at temperatures of 4 °C, 25 °C, and 37 °C by measuring OD at 450 nm (initial OD_450nm_ = 2) every month for a year and by evaluating the biological activity of the purified β-carotene as an antibacterial against *Staphylococcus aureus* ATCC (American Type Culture Collection) 25,913 at day 0 and after a year [28].

### Cytotoxicity of the purified β-carotene against human dermal fibroblasts (HDFs)

The HDFs were obtained from Sigma-Aldrich (St Louis, MO, USA) and grown in Dulbecco’s Modified Eagle Medium (DMEM) containing 10% fetal bovine serum through incubation in a humidified atmosphere supplemented with 5% CO_2_. Exponentially growing cells were seeded in 96-well culture plates, at densities of 3.500–5.500 cells per well, and incubated overnight to allow for cell attachment. After that, the cells were treated with diverse concentrations of the purified β-carotene (0, 31.25, 62.5, 125, 250, 500, and 1000 µg/ml), and DMSO was used as the solvent control. After incubation for 48 h, a volume of 20 µl of MTT solution was added and incubated for 4 h at 37 °C, and the formed formazan crystals were liquefied using DMSO (150 µl) for 10 min. Finally, the absorbance was measured at 490 nm via a spectrophotometer (PG, T80+, England). In addition, the cytotoxicity of the purified β-carotene against HDFs was examined using phase contrast microscopy. Briefly, the cells were treated with 240, 500, and 1000 µg/ml of β-carotene for 48 h, and the cells were examined using an inverted light microscope (Leica, Wetzlar, Germany) for the uncovering of the morphological changes [29].

### Isolates identification and antimicrobial susceptibility testing (AST)

*E. faecalis* isolates from infected urinary catheters were obtained from the microbiological laboratories of Tanta University Hospital, subcultured on blood agar, and identified by a series of biochemical tests and via the automated VITEK^®^ 2 system [30]. The AST via disc diffusion was performed according to the Clinical Laboratory Standards Institute (CLSI) guidelines using the following antimicrobial discs (Thermo Scientific™ Oxoid™): ampicillin (10 µg), ciprofloxacin (5 µg), erythromycin (15 µg), tetracycline (30 µg), and vancomycin (30 µg) [31].

### Antibacterial activity of the purified β-carotene

The antibacterial activity of purified β-carotene against *E. faecalis* isolates (*n* = 20) was tested using a well-diffusion technique, as described by Guan et al. (2023) with some modifications [32]. In brief, Mueller–Hinton (MH) agar plates were punctured with wells that were 6 mm in diameter. Each tested microbe (0.5 McFarland) was equally placed at a volume of 100 µl over the plate surface. The concentration of β-carotene in each well was 240 µg/ml. After the plates were incubated for 24 h at 37 °C, the diameter of the zone of inhibition (mm) that formed around each well was used to quantify the antibacterial activity. The negative control was 10% DMSO.

### The minimum inhibitory concentration (MIC) of β-carotene

The broth microdilution assay in MH broth was examined to determine the MIC values of β-carotene (1.875–240 µg/ml) against *E. faecalis* bacteria in accordance with the CLSI 2018 standard methodology. Following an overnight incubation period at 37 °C, the microplates were examined against a black background, and the broth’s turbidity was used for assessing the tested microorganism’s growth [33].

### Biofilm formation ability of *E. faecalis*

The capability of *E. faecalis* strains to form biofilms was evaluated using a crystal violet (CV) technique, as described by Kim et al. (2022) [34]. Briefly, a volume of 200 µl of prepared bacterial suspension in MH broth + 1% glucose (10^6^ colony forming unit (CFU)/ml) was transferred into a 96-well, flat-bottom microplate, and the plate was incubated under static conditions to allow biofilm formation at 37 °C. After 24 h of incubation, the excess media was removed and the wells were washed with 200 µl of phosphate-buffered saline (PBS) three times to remove unattached cells and then air dried. After that, the quantity of biofilm was measured using 0.1% CV as follows: 150 µl of methanol was added for 20 min to improve biofilm attachment; the plate was allowed to air dry, and 180 µl of 0.1% CV was added to each well for 15 min. The CV was discarded, and the remaining stain was removed by washing three times with sterile distilled water. Finally, 180 µl of 33% (v/v) glacial acetic acid was added to solubilize the embedded stain within biofilms, and the OD was measured at 595 nm using a microtiter reader (Sunrise™, TECAN, Switzerland). Each *E. faecalis* strain occupied three wells, and uninoculated MH broth served as a negative control. Based on the OD value of the negative control ODC (cut-off value), the resultant biofilm was categorized as follows:

$$\begin{aligned} {\text{OD}}\, \leqslant \,{\text{ODC}} &=\,{\text{biofilm is negative}} \hfill \\ {\text{ODC}}\, \leqslant \,{\text{OD}}\, \geqslant \,{\text{2 }} \times {\text{ ODC}} & =\,{\text{mildly positive for biofilm}} \hfill \\ {\text{2 }} \times {\text{ ODC}}\, \leqslant \,{\text{OD}}\, \geqslant \,{\text{4 }} \times {\text{ ODC}} &=\,{\text{moderately positive for biofilm}} \hfill \\ {\text{4 }} \times {\text{ ODC}}\, \leqslant \,{\text{OD}} & =\,{\text{intensely positive for biofilm}} \hfill \\ \end{aligned}$$

### Antibiofilm activity of β-carotene pigment against strong biofilm-forming *E. faecalis*

The effect of β-carotene on the biofilm formation by *E. faecalis* was evaluated, as described by Xiong et al. (2021) [35]. Bacterial suspension was prepared in MH broth supplemented with 1% glucose (10^8^ CFU/ml). After that, the bacterial suspension was 20-fold diluted to reach 5 × 10^6^ CFU/ml, then a volume of 180 µl of medium containing 0, 0.5 MIC, and 0.25 MIC of β-carotene and 20 µl of bacterial suspension was dispersed to each well of the microplate to obtain 5 × 10^5^ CFU/ml as a final concentration. The plate was incubated for 24 h at 37 °C, and the assay was performed in triplicate by measuring OD at 595 nm using a microtiter reader (Sunrise™, TECAN, Switzerland). The percentage of inhibition in biofilm formation was calculated by using the following formula:

$$~{\text{Percentage of inhibition}}~=\frac{{{\text{OD }}\left( {{\text{control}}} \right){\text{ }} - {\text{OD }}\left( {{\text{test}}} \right)}}{{{\text{OD }}\left( {{\text{control}}} \right)}} \times 100$$

### Antibiofilm activity of β-carotene against strong biofilm-forming *E. faecalis* using silicone catheter

The ability of β-carotene to inhibit biofilm formation was investigated on silicon catheters via staining with CV as described by Wongchai et al. (2024) [36]. A 50 mm-cut silicon catheter segments were submerged in β-carotene solution (0.5 MIC) for 1 h, then dried at room temperature for 24 h. The uncoated and pre-coated catheter segments were inoculated in a 24-well microplate containing a suspension of *E. faecalis* in TSB (10^7^ CFU/ml). After 24 h of incubation at 37 °C, the wells were washed with 0.85% NaCl, and the catheter segments were stained for 15 min with a 0.1% CV, then rinsed with 0.85% NaCl and resolved in 95% ethanol, and the biofilm inhibition was determined by measuring OD at 595 nm using a microtiter reader (Sunrise™, TECAN, Switzerland). The percentage of inhibition in biofilm formation was calculated as mentioned above.

### In situ visualization of the antibiofilm activity of β-carotene against strong biofilm-forming *E. faecalis*

#### Staining method using CV

The light microscopic analysis of CV-stained biofilms was utilized for observation of the effect of β-carotene on the biomass of *E. faecalis* biofilms, as detailed by Venkatramanan et al. (2020) [37]. Briefly, a volume of 2 ml of prepared *E. faecalis* suspension, 0.5 McFarland, in MH broth + 1% glucose (containing 0, 0.25 MIC, and 0.5 MIC of β-carotene) was transferred into a 6-well microtitre plate containing glass coverslips, and the plate was incubated in a static condition at 37 °C. After 24 h of incubation at 37 °C, glass coverslips, treated and untreated, had their unbound planktonic cells removed, rinsed with sterile distilled water (SDW), and stained with 0.1% CV. After twice washing excessive CV with SDW, the adhered biofilm and stained-glass coverslips were examined under a light microscope (LABOMED, CXL, USA).

#### Dual staining method

After the development of β-carotene-treated and untreated biofilms of *E. faecalis* as stated above, the biofilms were stained using Congo red and Maneval’s stain as prescribed by Manhas et al. (2024) [38]. The biofilms were gently washed with distilled water, then fixed with formaldehyde (4%) at room temperature for 30 min. The fixed biofilms were stained with Congo red stain (1%), air dried, and then stained for 10 min with Maneval’s stain. The excess stain was removed by washing, and the coverslips were dried. Finally, the biofilms were screened under a light microscope (LABOMED, CXL, USA) at 100x magnification.

#### Fluorescence staining method

The antibiofilm influence of the produced pigment against *E. faecalis* biofilm was further examined by confocal laser scanning microscopy (CLSM) after staining with acridine orange (AO) and propidium chloride (PI). In brief, the overnight incubated *E. faecalis* in MH broth + 1% glucose (containing 0, 0.25 MIC, and 0.5 MIC of β-carotene) was stained with 100 µmol AO/PI (300 µl) after removal of media and washing with PBS, then the biofilms were observed under the fluorescence microscope (DMi8; Leica Microsystems) [39].

### The antibiofilm mechanism of β-carotene against *E. faecalis*

The effect of β-carotene on gelatinase production was tested as prescribed by Seleem et al. (2021) for screening its antibiofilm mechanism [40]. Each tested strain of *E. faecalis* was cultured overnight in Luria Bertani (LB) broth with and without 0.25 and 0.5 MIC of β-carotene. The cultures were centrifuged and filtered through a 0.45 μm filter. The wells created in the gelatin agar plates (1.5% LB agar supplemented with 3% gelatin) were filled with 100 µl of sterile supernatants. Following a 24 h incubation period at 37 °C, 10 ml of Frazier’s solution was added to the plates, and the clear zones that developed around the wells were assessed.

### Determination of the *GelE* gene expression using quantitative reverse transcription PCR (qRT-PCR)

The mechanistic action of the produced β-carotene pigment against *E. faecalis*’s biofilm was investigated by evaluating the change in the expression of the *gelE* gene after treatment with 0.5 MIC of β-carotene using qRT-PCR. At first, the total ribonucleic acid (RNA) of untreated and β-carotene-treated samples was extracted using the kit’s instructions (Roche Diagnostic GmbH, Germany). The complementary DNA was synthesized by reverse transcription according to the manufacturer’s protocol (Promega, ImProm-II™ Reverse Transcription System). Finally, the qRT-PCR was performed using a reaction volume of 25 µl. The sequences of primers are presented in Table S2. The reaction conditions were 95 ˚C for 30 s, followed by 40 cycles of 95 ˚C for 5 s, 60 ˚C for 30 s, and 72 ˚C for 60 s using a Rotor-Gene Q device (Qiagen, USA). Lastly, the CT value of the *gelE* gene was normalized to the CT value of the * 16 s rRNA* gene (internal control), and the relative gene expression was determined using the 2^−ΔΔCT^ method [4, 41, 42].

### Molecular Docking of β-carotene with gelatinase protein

Using the online PubChem, the chemical structure of β-carotene was verified and obtained (https://pubchem.ncbi.nlm.nih.gov/compound/5280489). The gelatinase protein’s structure was retrieved in Protein Data Bank (PDB) format from UniProt (https://www.uniprot.org/uniprotkb/Q833V7/entry). The Molecular Operating Environment (MOE, 10.2008) software was used to carry out the binding interaction between β-carotene and the gelatinase protein. Water molecules and ligands not involved in the binding were removed, and the gelatinase protein was then prepared for docking analysis with MOE’s default protonate 3D methodology [43].

### Statistical analysis

The unpaired t test was performed using GraphPad Prism version 5.0. The analysis of variance (ANOVA) test was performed using Minitab 19.1 statistical software. The mean of three replicates ± standard deviation (SD) was used to represent the data. At a p-value of less than 0.05, differences in means were considered significant.

## Results

### Isolation of *M. luteus* from soil and screening of carotenoid pigment production

**Fig. 1:**
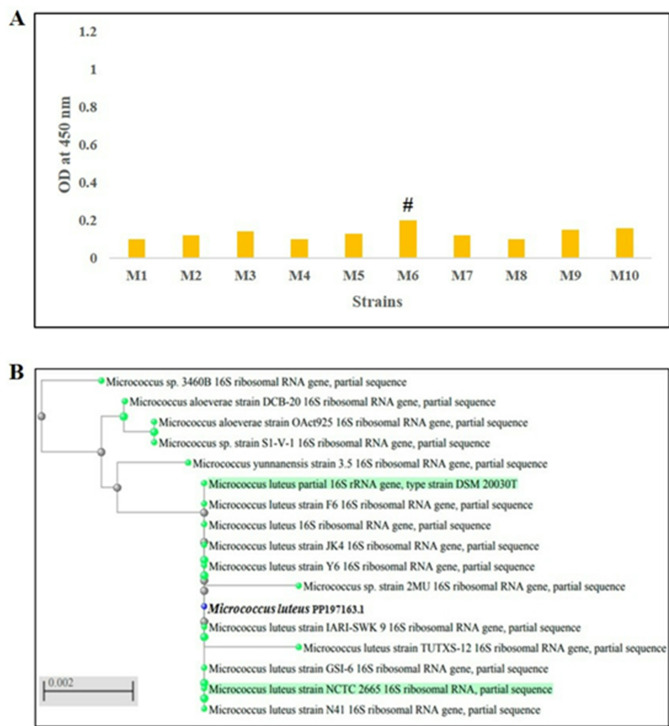
The OD values of methanolic extracts of carotenoid pigment from *M. luteus* isolates (*n* = 10) (**A**). Phylogenetic tree of *M. luteus* (M6 isolate) (accession number: PP197163) (**B**). M: *M. luteus*. ^#^indicates the highest producer isolate

A total of 33 soil samples were investigated for yellow-pigmented colonies of *M. luteus*, which were identified by their microscopic features, biochemical examinations, and MALDI-TOF MS. Only 10 yellow-pigmented isolates showed Gram-positive cocci that had the characteristic tetrad arrangement of *M. luteus* after Gram staining (Fig. S1) and the same biochemical test profile of *M. luteus* (positive catalase, positive oxidase, negative glucose fermentation, and non-motile bacteria). The identification of the isolates as *M. luteus* was confirmed by MALDI-TOF MS. The isolates’ ability to produce a carotenoid pigment was screened using methanol for pigment solubilization. After that, we measured the OD at 450 nm for each methanolic extract, as shown in Fig. 1A. The data showed that the OD measurements of the methanolic extracts ranged from 0.1 to 0.2. The isolates M6, M9, and M10 recorded ODs greater than 0.15, while the ODs of the remaining isolates were less than 0.15. The highest carotenoid-producing isolate was M6 (OD = 2), followed by M10 isolate (OD = 0.16), then M9 isolate (OD = 0.15).

#### Molecular characterization of the highest producer strain

By sequencing the *16 S rRNA* gene, the most productive strain (M6) was molecularly characterized, displaying 99.9% similarity with *M. luteus*, and the resultant sequence was added to the database of GenBank (accession number of PP197163). The degree of closeness between our sequenced strain and the other species of *Micrococcus* species was illustrated in the phylogenetic tree below (Fig. 1B).

#### Evaluation of the influence of physico-chemical parameters on carotenoid production using OFAT experiments

**Fig. 2:**
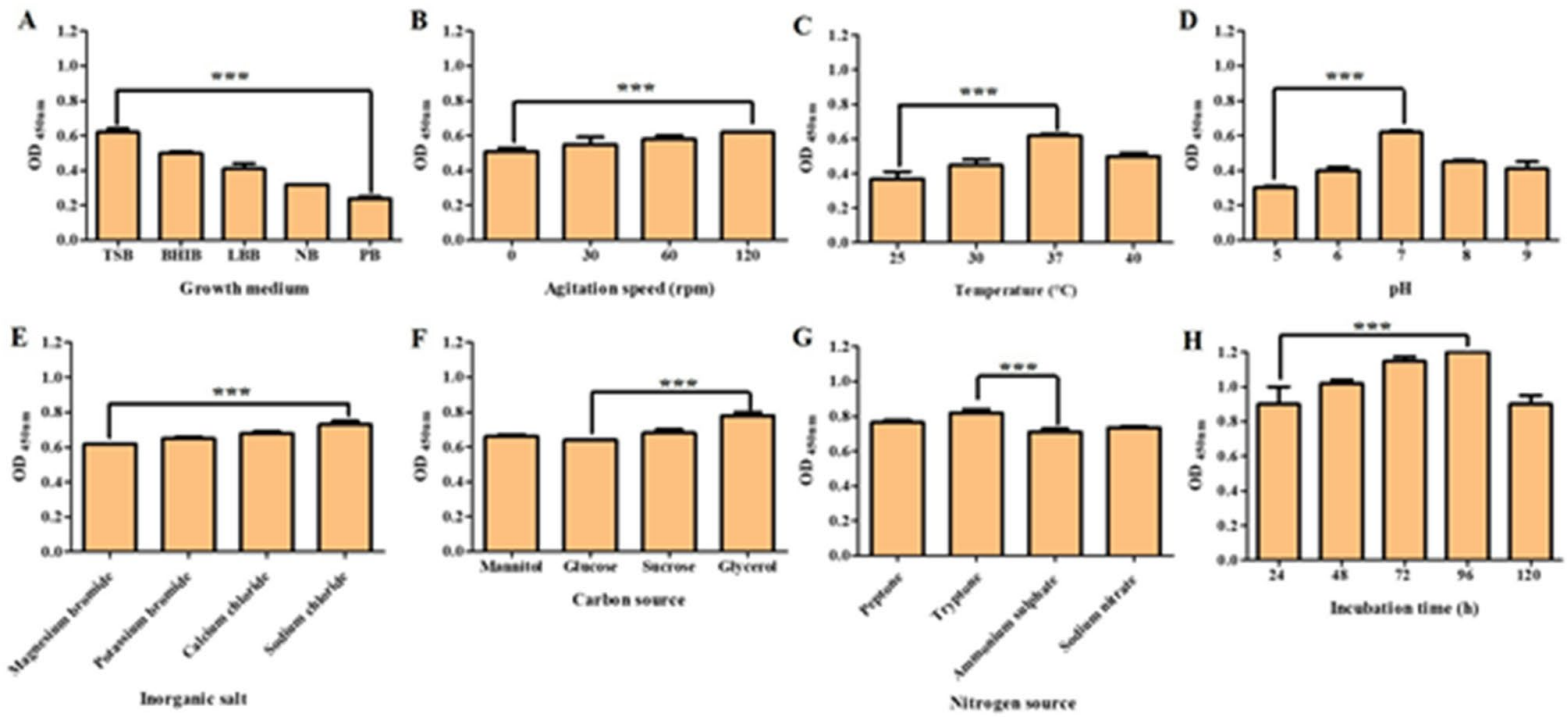
Results of OFAT experiments. The effect of growth medium on carotenoid production (**A**). The effect of agitation on carotenoid production (**B**). The effect of temperature on carotenoid production (**C**). The effect of pH on carotenoid production (**D**). The effect of inorganic salt on carotenoid production (**E**). The effect of carbon source on carotenoid production (**F**). The effect of nitrogen source on carotenoid production (**G**). The effect of incubation time on carotenoid production (**H**). Each bar represents the average values of triplicate measurements, and the asterisk represents the statistical difference at *P* < 0.0001

**Fig. 3:**
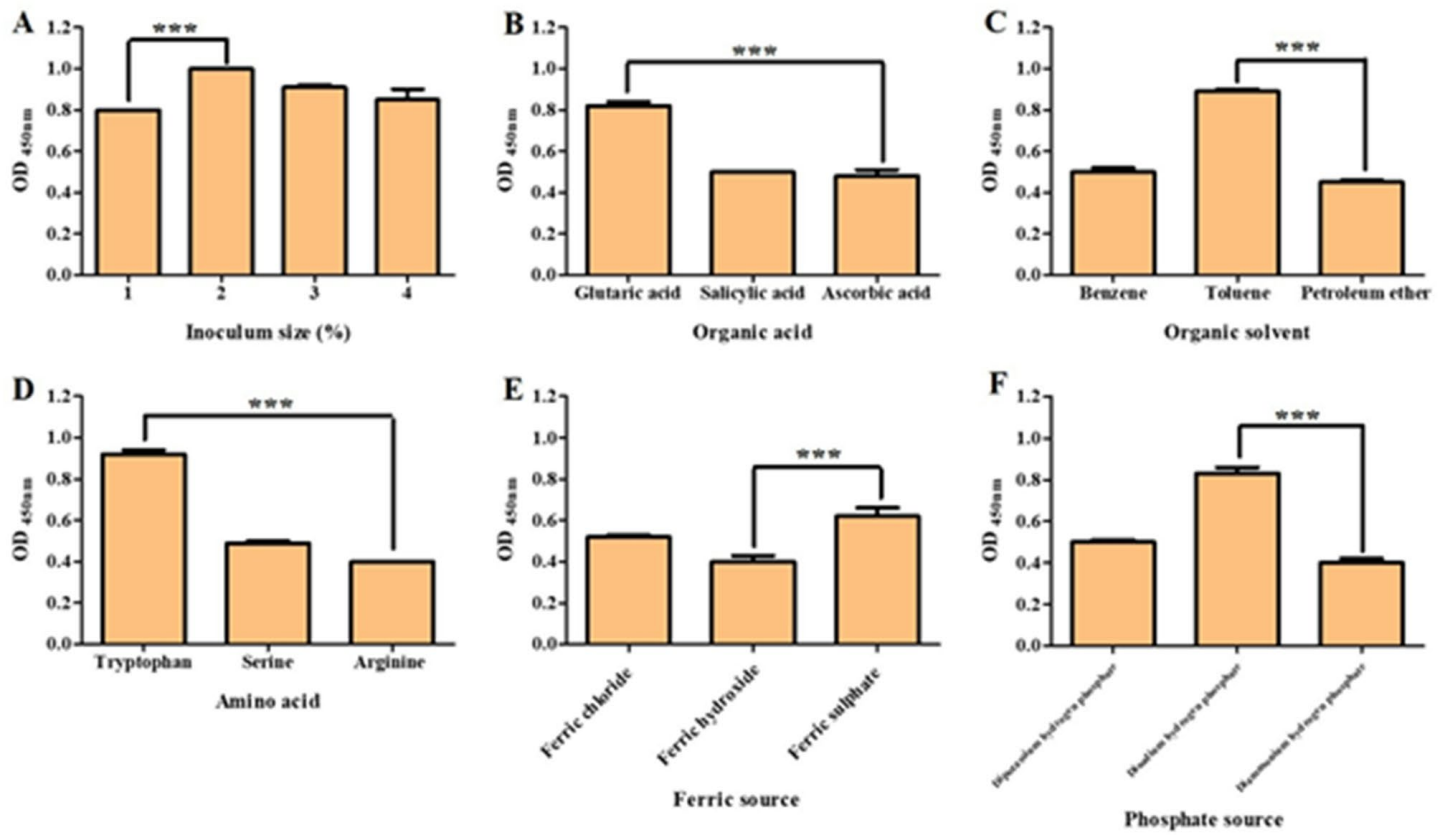
Results of OFAT experiments. The effect of IS on carotenoid production (**A**). The effect of organic acid on carotenoid production (**B**). The effect of organic solvent on carotenoid production (**C**). The effect of amino acid on carotenoid production (**D**). The effect of ferric source on carotenoid production (**E**). The effect of phosphate source on carotenoid production (**F**). Each bar represents the average values of triplicate measurements, and the asterisk represents the statistical difference at *P* < 0.0001

The effects of physical and chemical factors affecting pigment production, such as growth medium, agitation, temperature, pH, incubation period, IS, nitrogen source, carbon source, inorganic salt, organic acid, organic solvent, amino acid, ferric iron, and phosphate, were examined (Figs. 2 and 3). The results showed that there was a difference in carotenoid pigment production among the examined media, where the best medium was TSB, with an increase in carotenoid production by 158.3% compared to PB. By the examination of the effect of agitation on production ability, we found that an agitation speed of 120 rpm increased production by 21.5% compared to the static state. After evaluating the carotenoid pigment yield at different temperatures, we observed that the incubation at 37 °C was the best, with an increase in carotenoid production by 67.5% compared to incubation at 25 °C. The capacity of carotenoid production was screened at various pH values, and the results showed that the pigment yield at pH 7 was greater than yields recovered at other pH values, with an increase in the production of the carotenoid pigment by 106.6%. The incubation time was investigated, and the data showed that the pigment yield was increased after 96 h of incubation by 33.3% compared to 24 h of incubation. Also, it was found that the best IS for pigment production was 2%, which increased carotenoid pigment production by 25% compared to an IS of 1%. The pigment yields were measured after adding various inorganic salts, and the data showed that the addition of sodium chloride was optimal, as the pigment production was increased by 17.7% compared to magnesium bromide. The effect of adding different amino acids revealed that the addition of tryptophan increased pigment production by 130% compared to arginine. The impact of the carbon source was tested, and the data showed that adding glycerol increased carotenoid pigment production by 21.8% compared to glucose. The influence of several nitrogen sources was also examined, and it was found that adding tryptone led to an increase in carotenoid pigment production by 15.5% compared to sodium nitrate. Incubation with glutaric acid increased pigment production by 70.8% compared to ascorbic acid. In addition, incubation with toluene enhanced the production of pigment by 97.7% compared to petroleum ether. The addition of ferric sulphate increased pigment production by 55% compared to ferric hydroxide. Lastly, the addition of disodium hydrogen phosphate augmented the production of pigment by 107.5% compared to diammonium hydrogen phosphate.

#### Determination of upstream process parameters using PBD and RSM

**Fig. 4:**
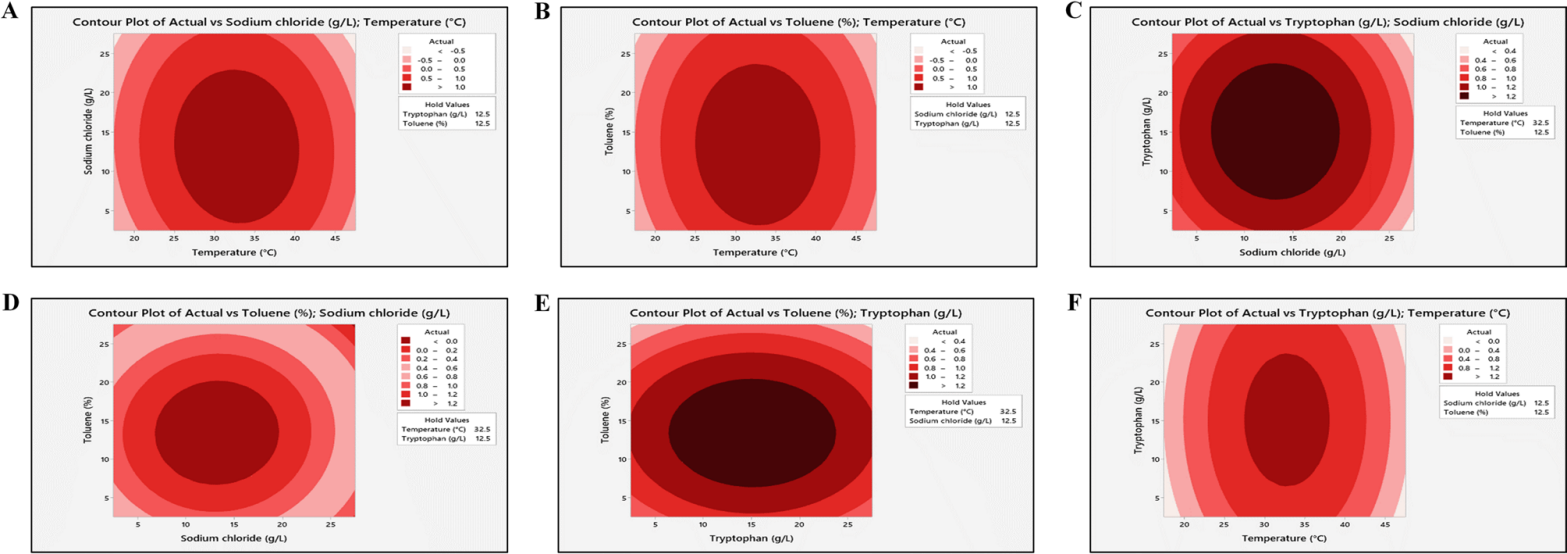
Results of RSM design. The 2D contour plots of actual vs. sodium chloride; temperature (**A**), actual vs. toluene; temperature (**B**), actual vs. tryptophan; sodium chloride (**C**), actual vs. toluene; sodium chloride (**D**), actual vs. toluene; tryptophan (**E**), and actual vs. tryptophan; temperature (**F**)

To determine the primary elements that significantly affected the M6 isolate’s ability to produce carotenoid pigment, a PBD was carried out by means of Minitab software (Table S3). Based on the results provided by the Pareto chart (Fig. S2), temperature, sodium chloride, tryptophan, and toluene were recognized as the topmost factors affecting the production of carotenoid pigment (standardized effect > 2.447). Using temperature, sodium chloride, tryptophan, and toluene as investigational factors and the OD at 450 nm as the response value, a CCD was created via Minitab software. The results of the established CCD are shown in Table 1. According to the results of the regression model (Table S4), items tryptophan, toluene, temperature (°C)*temperature (°C), sodium chloride (g/l)*sodium chloride (g/l), tryptophan (g/l)*tryptophan (g/l), and toluene (%)*toluene (%) had an extremely substantial outcome on actual values (*P* = 0.000). The good fit of the model was proved by the resultant R-squared = 0.9948, which meant that 99.48% of the results after each run could be predicted employing the model (> 90% is usually desired), signifying its reliability. The two-dimensional contour plots of the intensity of the carotenoid pigment were plotted, illustrating the impact of independent factors on the value of the response (OD at 450 nm). In contour plots, higher responses were categorized by the darker areas, as shown in Fig. 4. In conclusion, the optimal conditions for enhancing the production of carotenoid via M6 isolate were TSB medium, agitation speed of 120 rpm, temperature of 32.5 °C, pH = 7, incubation time of 96 h, 2% IS, sodium chloride (12.5 g/l), tryptophan (12.5 g/l), glutaric acid (5 g/l), toluene (12.5%), and disodium hydrogen phosphate (5 g/l).

**Table 1:** Result of CCD experiment

Run	Temperature (°C)	Sodium chloride (g/l)	Tryptophan (g/l)	Toluene (%)	Actual	Predicted
1	32.5	12.5	12.5	12.5	1.34	1.347
2	32.5	12.5	12.5	12.5	1.34	1.34
3	25	20	5	20	0.5	0.52
4	32.5	12.5	12.5	27.5	0.72	0.687
5	17.5	12.5	12.5	12.5	0.081	−0.004
6	25	5	5	20	0.36	0.377
7	40	20	5	20	0.5	0.503
8	32.5	27.5	12.5	12.5	0.639	0.594
9	32.5	12.5	12.5	12.5	1.34	1.347
10	40	20	20	5	0.57	0.581
**11**	**32.5**	**12.5**	**12.5**	**12.5**	**1.35**	**1.347**
12	32.5	2.5	12.5	12.5	0.95	0.927
13	25	5	5	5	0.25	0.272
14	25	20	20	5	0.54	0.561
15	32.5	12.5	2.5	12.5	1.06	1.014
16	40	20	20	20	0.66	0.661
17	40	20	5	5	0.42	0.413
18	47.5	12.5	12.5	12.5	0.06	0.091
19	40	5	20	20	0.64	0.627
20	40	5	5	5	0.4	0.393
21	25	5	20	5	0.43	0.456
22	40	5	20	5	0.58	0.577
23	32.5	12.5	12.5	12.5	1.34	1.347
24	25	5	20	20	0.52	0.551
25	32.5	12.5	12.5	12.5	1.34	1.347
26	25	20	5	5	0.35	0.392
27	32.5	12.5	27.5	12.5	1.06	1.025
28	32.5	12.5	12.5	12.5	1.32	1.347
29	40	5	5	20	0.45	0.453
30	25	20	20	20	0.65	0.686
31	32.5	12.5	12.5	2.5	1	0.951

The bold symbol indicates the run conditions with the highest yield

#### Submerged fermentation model validation and extraction of carotenoid pigment from M6 isolate

**Fig. 5:**
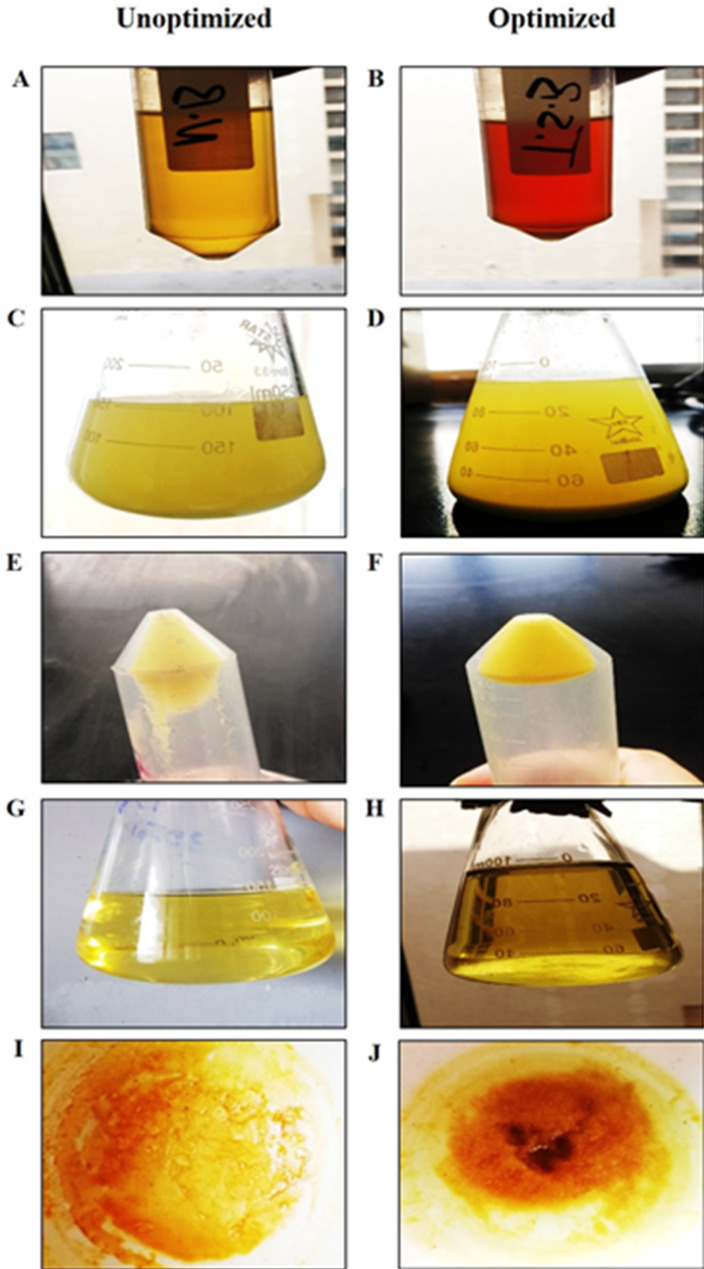
The extraction procedures of carotenoid pigment from M6 isolate after incubation in unoptimized and optimized conditions. Incubation of M6 isolate in NB and TSB media (**A**,** B**). Methanolic extraction of carotenoid pigment (**C**,** D**). The formed pellet after the first extraction step of the carotenoid pigment (**E**,** F**). The whole methanolic extract of carotenoid pigment (**G**,** H**). The carotenoid powder was formed after evaporation of the solvent at 70 °C (**I**,** J**)

After the incubation of the M6 isolate in optimized and unoptimized conditions, the pigment was extracted using methanol at 60 °C, which was indicated by the yellow colorization of the methanol solvent. After centrifugation to obtain cell-free pigmented methanol, the formed pellet was resuspended in methanol to extract the remaining yellow pigment until the pellet’s color turned white, representing the full extraction of the carotenoid. The produced pigments were dried and weighed, and it was found that the amount of pigment in unoptimized conditions was 50 mg; on the other hand, the amount of pigment after using optimized conditions was 300 mg (6-fold enhancement) (Fig. 5).

#### Purification and characterization of the extracted carotenoid pigment

**Fig. 6:**
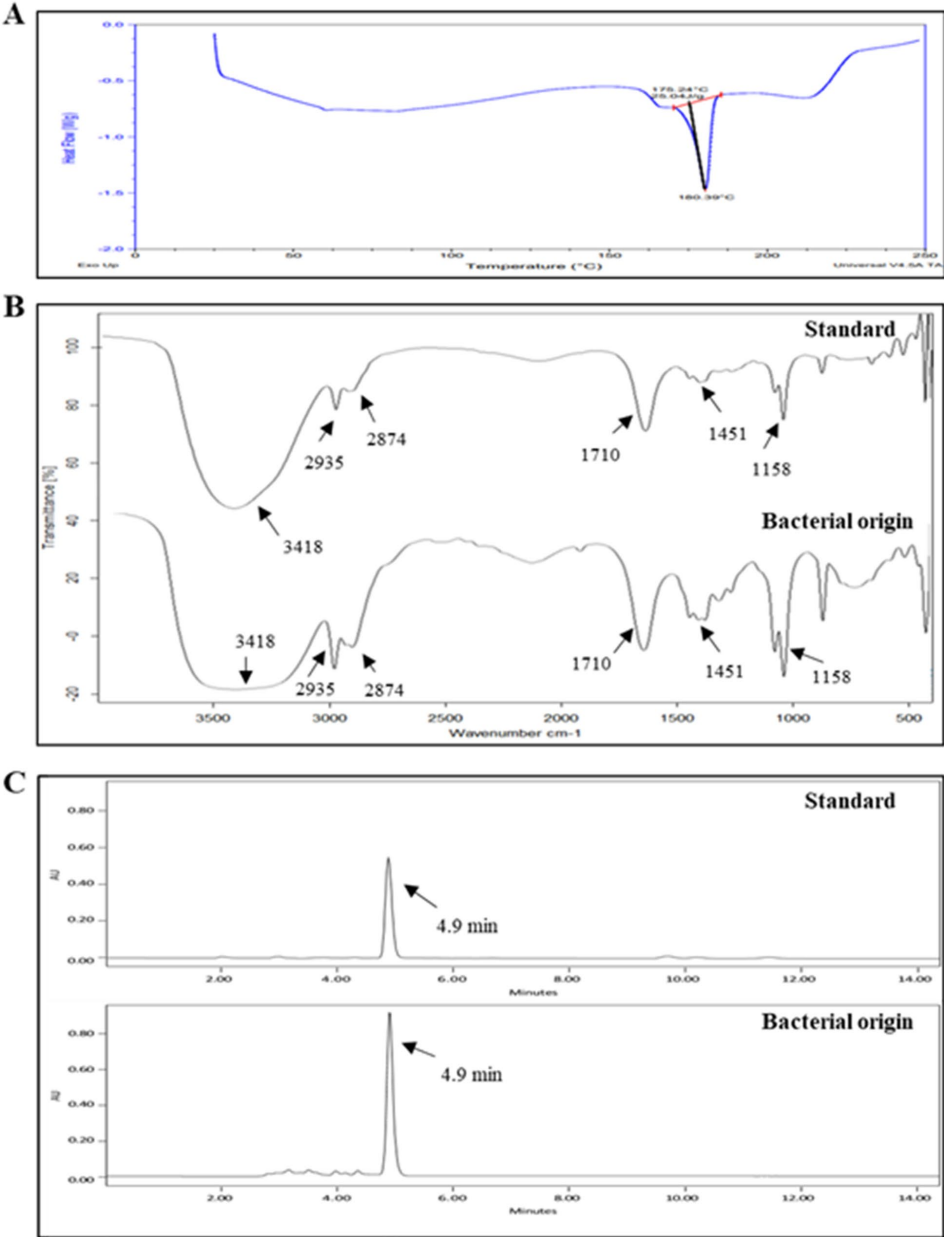
Characterization of the purified pigment. DSC curve showed the characteristic endothermic peak of β-carotene at 180 °C (**A**). FTIR spectra of the β-carotene standard (Sigma-Aldrich) and the M6 isolate’s purified β-carotene (**B**). HPLC chromatograms of standard β-carotene and M6 isolate’s β-carotene (**C**)

The extracted pigment was purified by silica gel column chromatography and characterized. Based on trial and error in developing a TLC method for separating the M6 isolate’s pigment, acetone and *n*-hexane (6:4) were utilized as a solvent system, and the value of the retardation factor (R_f_) was measured. The TLC plate showed a single spot with an R_f_ value equal to 0.89, indicating the high purity of the extracted pigment (Fig. S3). The melting point of a sample is often used to determine its purity; therefore, the purity of the extracted pigment was confirmed using DSC, and the resulting melting curve was examined. As shown in Fig. 6A, the melting curve showed a sharp peak at 180.39 °C, which is the characteristic melting point of β-carotene. Furthermore, the resultant curves from FTIR for the extracted β-carotene pigment and standard β-carotene showed approximately the same vibration bands at 3418, 2935, 2874, 1710, 1451, and 1158 cm^−1^ (Fig. 6B). In addition, after HPLC analysis, the extracted pigment and standard β-carotene displayed one peak at a similar retention time (4.9 min) (Fig. 6C), confirming that the extracted pigment from the M6 isolate was β-carotene.

#### Stability study of the extracted carotenoid pigment

**Fig. 7:**
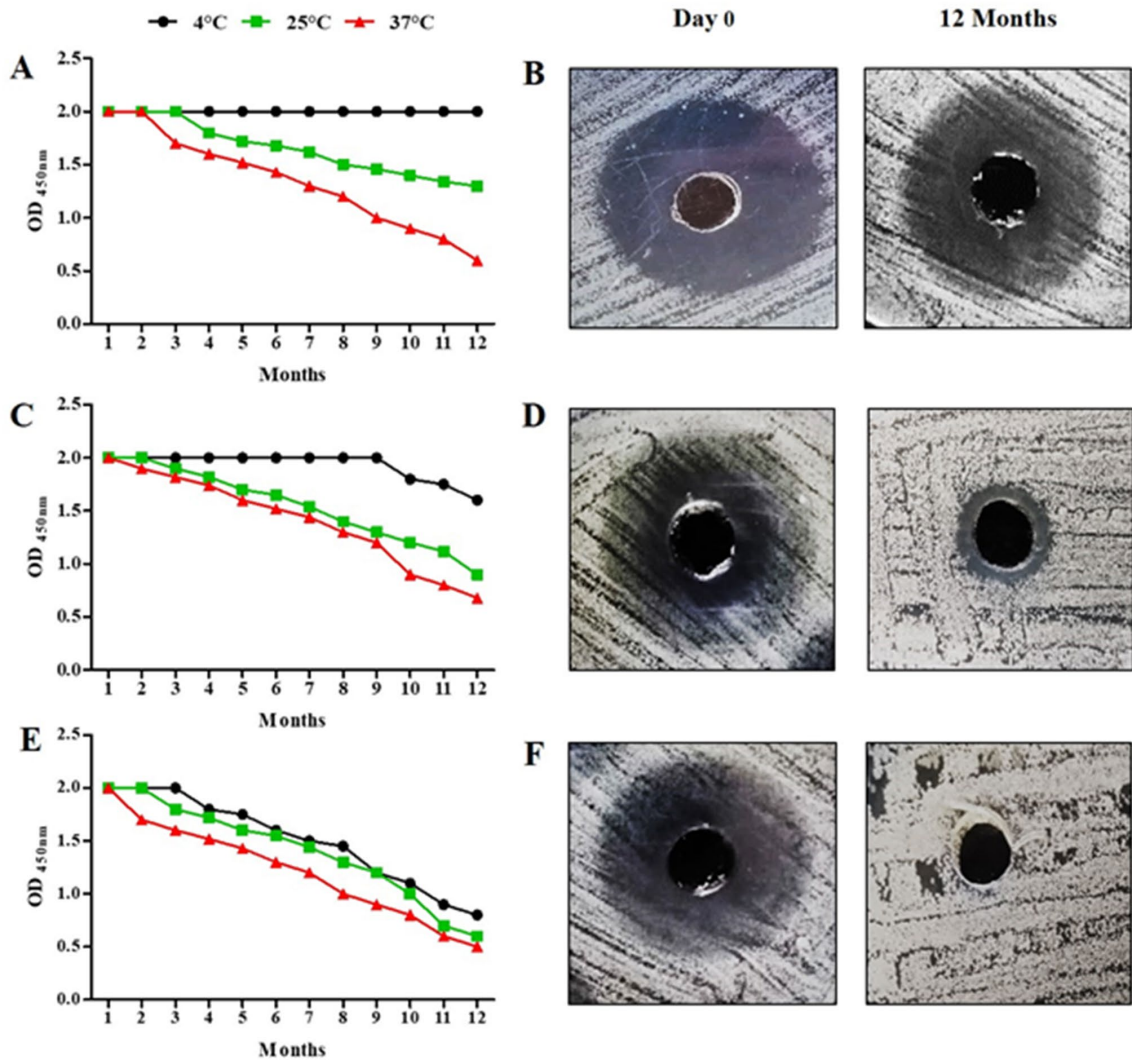
Stability study for the produced β-carotene for a year in DMSO, ethanol, and methanol at different temperatures of 4 °C, 25 °C, and 37 °C. OD measurements for the produced β-carotene dissolved in DMSO (**A**), ethanol (**C**), and methanol (**E**). The inhibition zones at day 0 and after a year for the produced β-carotene in DMSO (**B**), ethanol (**D**), and methanol (**F**)

To identify the ideal storage conditions for future use, stability experiments were conducted to ascertain the impact of temperature and solvent on the produced β-carotene. For a year, we assessed the OD at 450 nm every month after dissolving the same quantity of β-carotene in several solvents, including DMSO, methanol, and ethanol. Additionally, the stability was investigated by measuring the inhibition zone against *S. aureus* ATCC 25,913 at day 0 and after a year. The stable β-carotene exhibited the same OD value and inhibition zone, whereas the OD and inhibition zone of degraded β-carotene decreased. The best storage conditions were storage of β-carotene in DMSO at 4 °C (β-carotene stable for up to a year). In contrast, storage in DMSO at 25 °C and DMSO at 37 °C remained stable for 3 months and 2 months, respectively. The storage in ethanol at 4 °C, 25 °C, and 37 °C remained stable for 9 months, 2 months, and 1 month, respectively. The storage in methanol at 4 °C, 25 °C, and 37 °C remained stable for 3 months, 2 months, and 1 month, respectively (Fig. 7).

#### Cytotoxicity of the extracted β-carotene on HDFs using MTT assay and phase-contrast microscopy

**Fig. 8:**
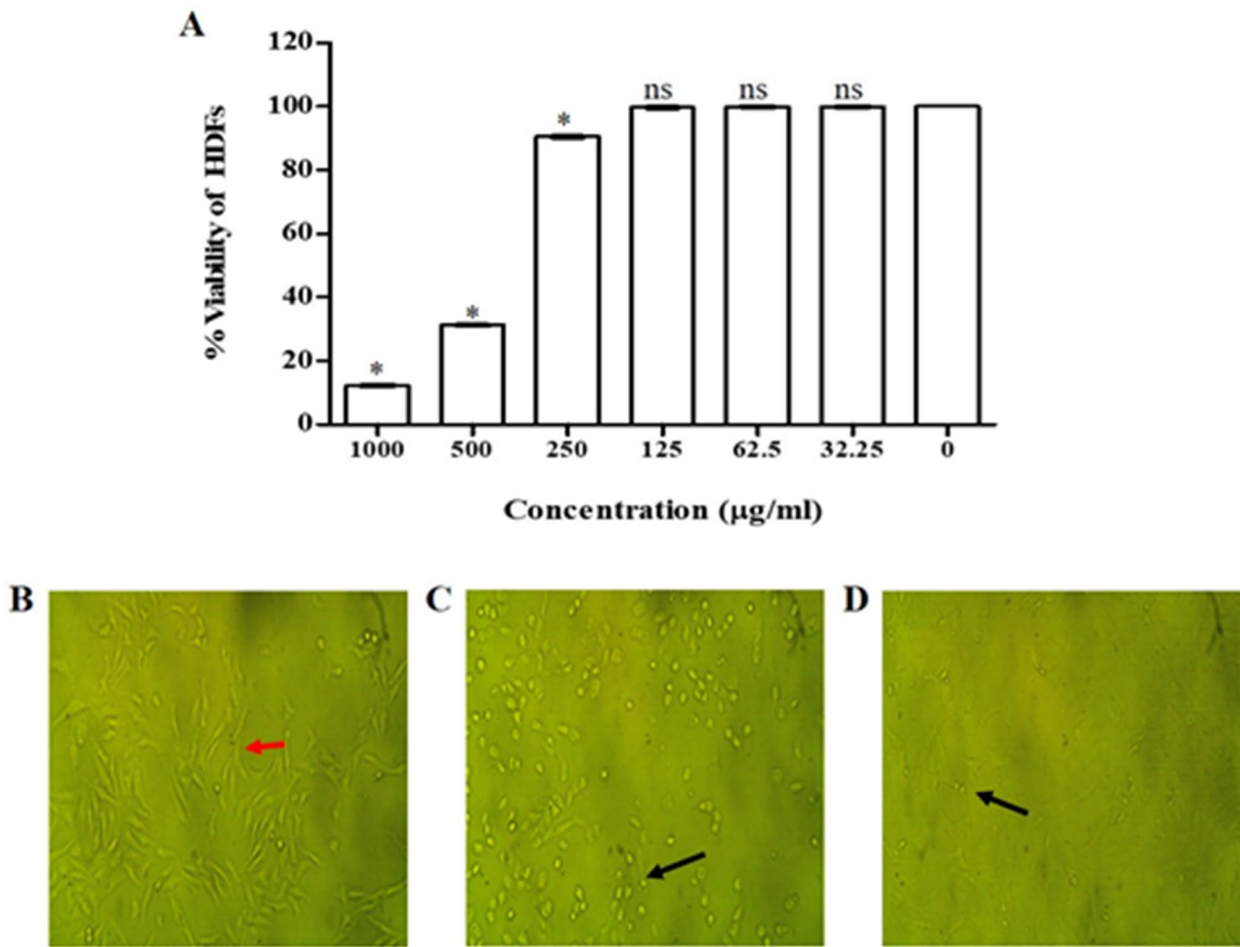
Cytotoxicity of M6 isolate’s β-carotene pigment on HDFs. The bar chart represents the percentages of reduction in the viability of HDFs after treatment with different concentrations of β-carotene (**A**). The HDFs showed typical cell characteristics (Red arrow) when treated with 240 µg/ml of β-carotene (**B**). Morphological changes of HDFs after treatment with 500 µg/ml (**C**) and 1000 µg/ml (**D**) of the extracted β-carotene, showing cell rounding and shrinkage (Black arrow). Each bar represents the average values of triplicate measurements, and the asterisk represents the statistical difference at *P* < 0.05. ns; nonsignificant

Cytotoxicity of the β-carotene produced by the M6 isolate was determined using the MTT assay on HDFs before the evaluation of its biological activities. The results showed that the difference in the viability of HDFs was insignificant after treatment with β-carotene at concentrations less than 250 µg/ml. In contrast, the viability of HDFs after treatment with β-carotene, at concentrations higher than 250 µg/ml, significantly declined (*P* < 0.05), with IC_50_ equal to 542.7 µg/ml (Fig. 8A). Subsequently, safe concentrations of β-carotene pigment, less than 250 µg/ml, were used in subsequent analysis. In addition, the cytotoxic effect of β-carotene on HDFs was confirmed by screening the morphological changes using phase-contrast microscopy. As shown in Fig. 8B–D, the number of HDFs decreased after 48 h of treatment with 500 and 1000 µg/ml of β-carotene, manifesting signs of apoptosis, such as rounding and shrinking of the cells. In contrast to 240 µg/ml-treated cells showed a higher number of cells and typical cell characteristics without any discernible changes.

#### Isolate identification and AST

A total of 20 isolates were identified as *E. faecalis* according to results of biochemical tests, which were confirmed via the automated VITEK 2 system (Table S5). The data from AST revealed that all isolates were MDR (not susceptible to at least one agent in three or more antimicrobial classes); 5 isolates were resistant to ampicillin, 19 isolates were resistant to ciprofloxacin, and all isolates were resistant to erythromycin and tetracycline. In contrast, all isolates were sensitive to vancomycin (Table S6).

#### Antibacterial activity against *E. faecalis* and MIC determination

**Fig. 9:**
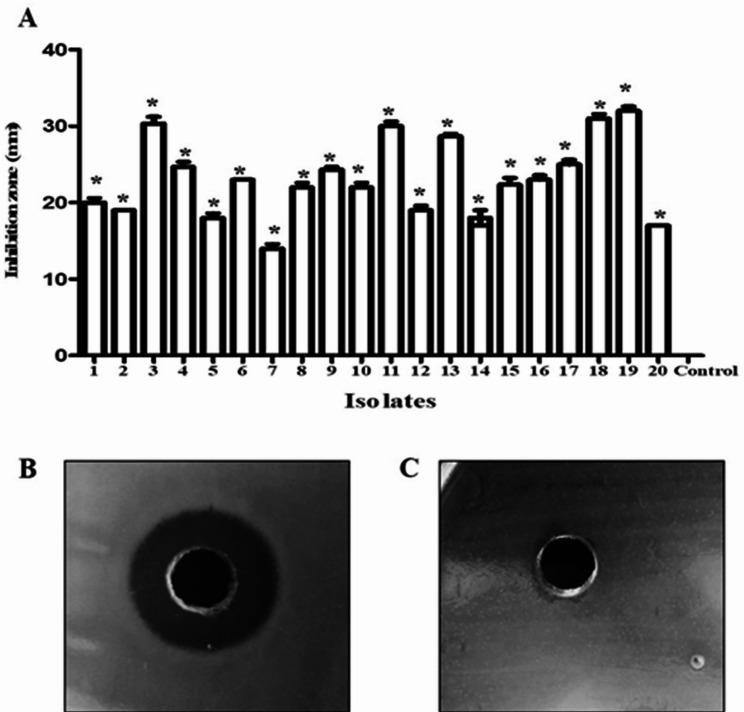
Antibacterial activity of β-carotene (240 µg/ml) against *E. faecalis*. The bar chart represents the inhibition zones of β-carotene against *E. faecalis* isolates (**A**). A representative plate showing the produced inhibition zones against *E. faecalis* (**B**). The negative control (10% DMSO) demonstrated no inhibition zone (**C**). Each bar represents the average value of triplicate measurements, and the asterisk indicates a statistically significant difference at *P* < 0.05 compared to the control

The antibacterial activity of β-carotene was investigated against 20 *E. faecalis* isolates using a well diffusion assay. The growth of all *E. faecalis* isolates was significantly (*P* < 0.05) inhibited by β-carotene, and the resultant inhibition zones ranged from 13 mm to 32 mm (Fig. 9). MICs of β-carotene against *E. faecalis* isolates were determined by the microdilution technique, and the data showed that the MICs of β-carotene ranged from 3.75 to 30 µg/ml (Table 2).

**Table 2:** MICs of the extracted β-carotene against *E. faecalis* strains

Isolate	MIC value	Isolate	MIC value	Isolate	MIC value
1	15	8	7.5	15	7.5
2	15	9	7.5	16	7.5
3	3.75	10	7.5	17	7.5
4	7.5	11	3.75	18	3.75
5	15	12	15	19	3.75
6	7.5	13	3.75	20	15
7	30	14	15		

#### Biofilm-forming capacity of MDR *E. faecalis*

The biofilm formation ability of *E. faecalis* (*n* = 20) was determined using the CV staining technique, and the OD was measured at 595 nm. The measured ODs of isolates 1, 2, 3, 4, 12, 13, 14, and 20 were greater than 4 × ODC (> 0.4), indicating their intense biofilm formation capability, as listed in Table S7. In contrast, the remaining isolates showed a moderate to weak biofilm formation capacity.

#### Inhibition of biofilm formation by MDR *E. faecalis* via the extracted β-carotene

**Fig. 10:**
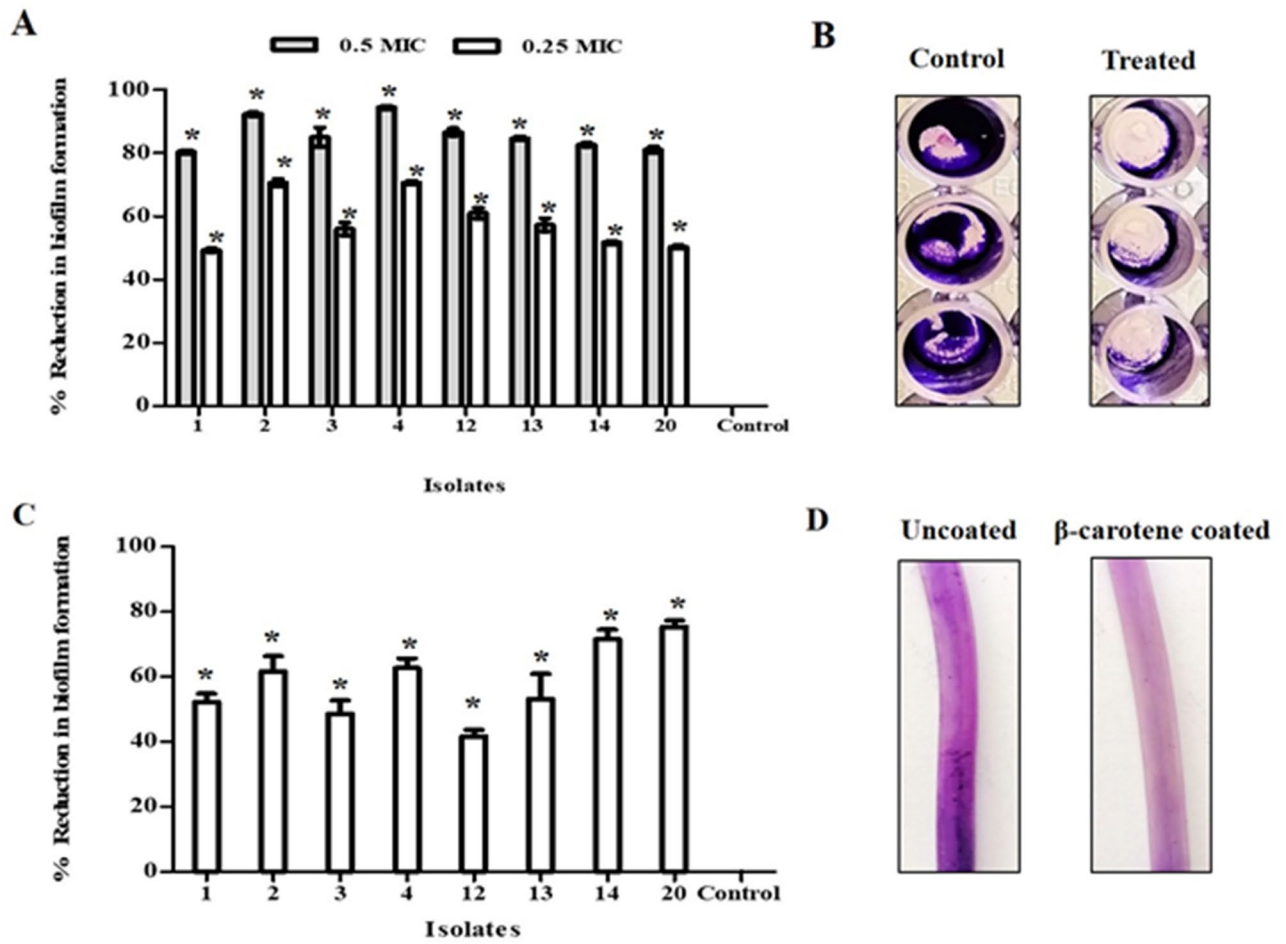
Biofilm inhibition activity of β-carotene against *E. faecalis.* The bar chart represents a dose-dependent biofilm-inhibitory activity of β-carotene (0.25 and 0.5 MICs) against *E. faecalis* using a microtiter plate assay (**A**). A representative image of microtiter plate results displaying a reduction in biofilm biomass after treatment with β-carotene (0.5 MIC) (**B**). The percentage of reduction in biofilm formation by *E. faecalis* on a silicon catheter (**C**). Segments of silicon catheter showed the effect of β-carotene coating in the reduction of biofilm formation by *E. faecalis* (**D**). Each bar represents the average value of triplicate measurements, and the asterisk indicates a statistically significant difference at *P* < 0.05 compared to the control

The activity of β-carotene on biofilm formation by strong biofilm-forming *E. faecalis* (*n* = 8) was tested using CV staining, and the percentages of reduction in the measured ODs at 595 nm were calculated. The biofilm formation by all tested *E. faecalis* was significantly (*P* < 0.05) reduced by β-carotene application in a dose-dependent manner, and the percentage of biofilm inhibition ranged from 49 to 70% for 0.25 MIC of β-carotene, where it ranged from 81 to 94% for 0.5 MIC of β-carotene, as presented in Fig. 10A, B.

#### Biofilm formation on silicone catheters was prevented by β-carotene

Using a silicone catheter as a model for medical devices, the inhibitory efficacy of β-carotene against the formation of biofilms by *E. faecalis* was assessed. When stained with CV, coated catheters showed a thinner biofilm because the β-carotene coating prevented sessile cells from adhering to the catheter’s surface. On the other hand, the uncoated catheters showed thick biofilms. As illustrated in Fig. 10C, D, the percentages of biofilm inhibition on coated catheters varied from 41.6 to 75.3%. These results supported β-carotene’s capacity to inhibit the production of *E. faecalis* biofilms.

#### In situ visualization of antibiofilm activity of β-carotene via light microscope and confocal laser scanning microscope

**Fig. 11:**
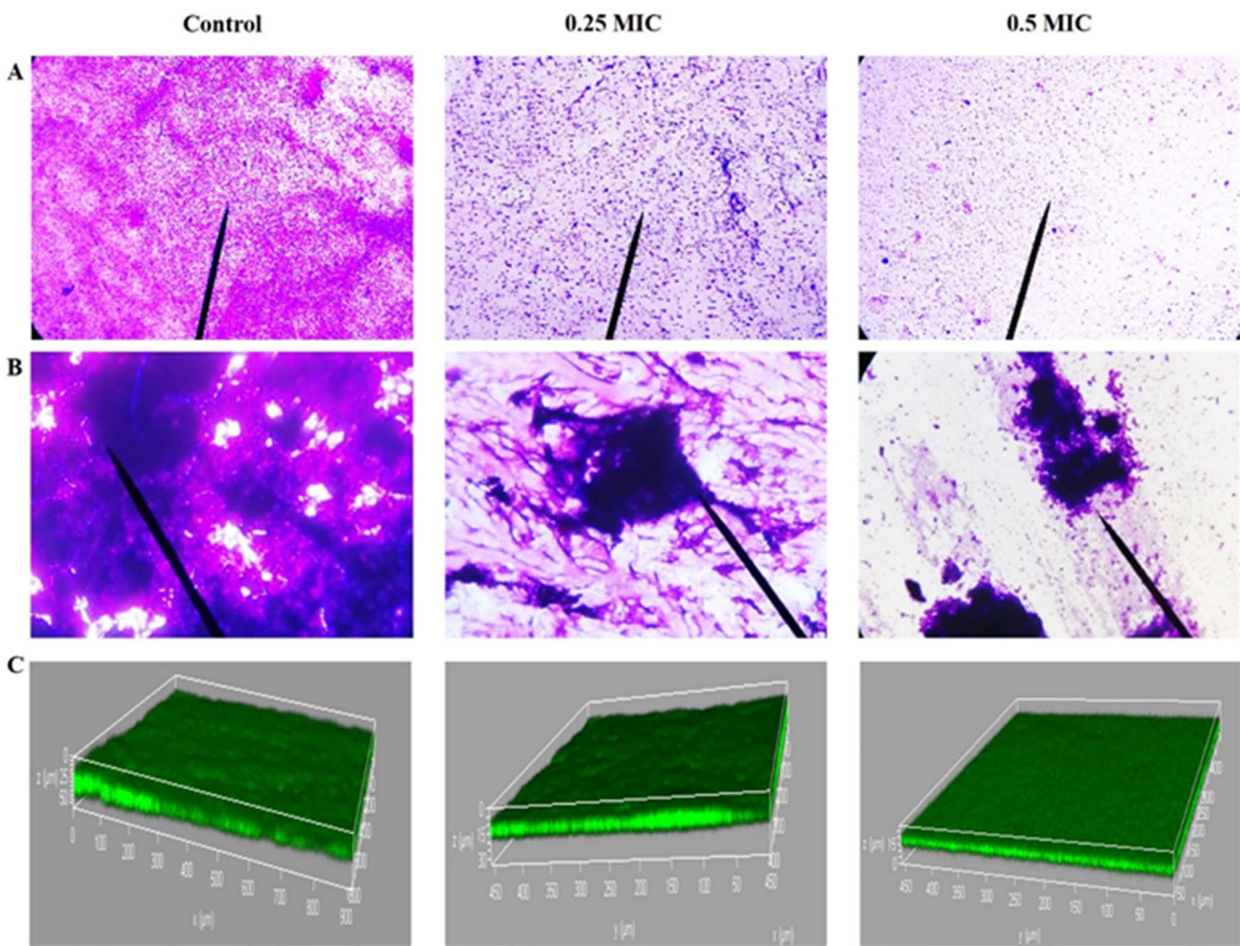
In situ visualization of antibiofilm activity of β-carotene against *E. faecalis* after treatment with 0.25 and 0.5 MICs. The intensity of CV-stained biofilms was reduced after treatment with β-carotene (**A**). The dual staining technique showed a dense blue colored matrix and numerous magenta-colored sessile cells in the control compared to β-carotene-treated biofilms (**B**). The three-dimensional images of CLSM show the reduction in biofilm thickness and biomass (green fluorescence) after treatment with β-carotene (**C**)

Light microscopy examination of CV-stained biofilms emphasized the antibiofilm activity of the extracted β-carotene (0.5 and 0.25 MICs) against strongly biofilm-forming *E. faecalis*, showing a reduction in the number of cells within the biofilm. In contrast, light microscopy images of control plates showed the presence of a dense complex structure of biofilm (Fig. 11A). Additionally, when the structure of the biofilm was visualised using the dual staining method, the biofilm’s polysaccharide matrix became dark blue. To differentiate the sessile cells from the biofilm matrix, acid fuchsin was used to color the cells magenta-red. As illustrated in Fig. 11B, the pretreatment with β-carotene (0.5 and 0.25 MICs) inhibited biofilm formation by *E. faecalis*, resulting in an immature biofilm with a less dense polysaccharide matrix and fewer sessile cells embedded within the matrix, compared to untreated biofilms. The antibiofilm effect of β-carotene on *E. faecalis* biofilms was verified by CLSM examination. The three-dimensional images of control and treated biofilms showed a significant (*P* < 0.05) reduction in biomass and thickness of the biofilms after treatment with 0.5 MIC (biofilm thickness = 15 μm) and 0.25 MIC (biofilm thickness = 30 μm) of β-carotene. On the other hand, the control showed a robust biofilm biomass (biofilm thickness = 50 μm) (Fig. 11C).

#### Antibiofilm activity of β-carotene was dependent on Inhibition of gelatinase production by E. faecalis

**Fig. 12:**
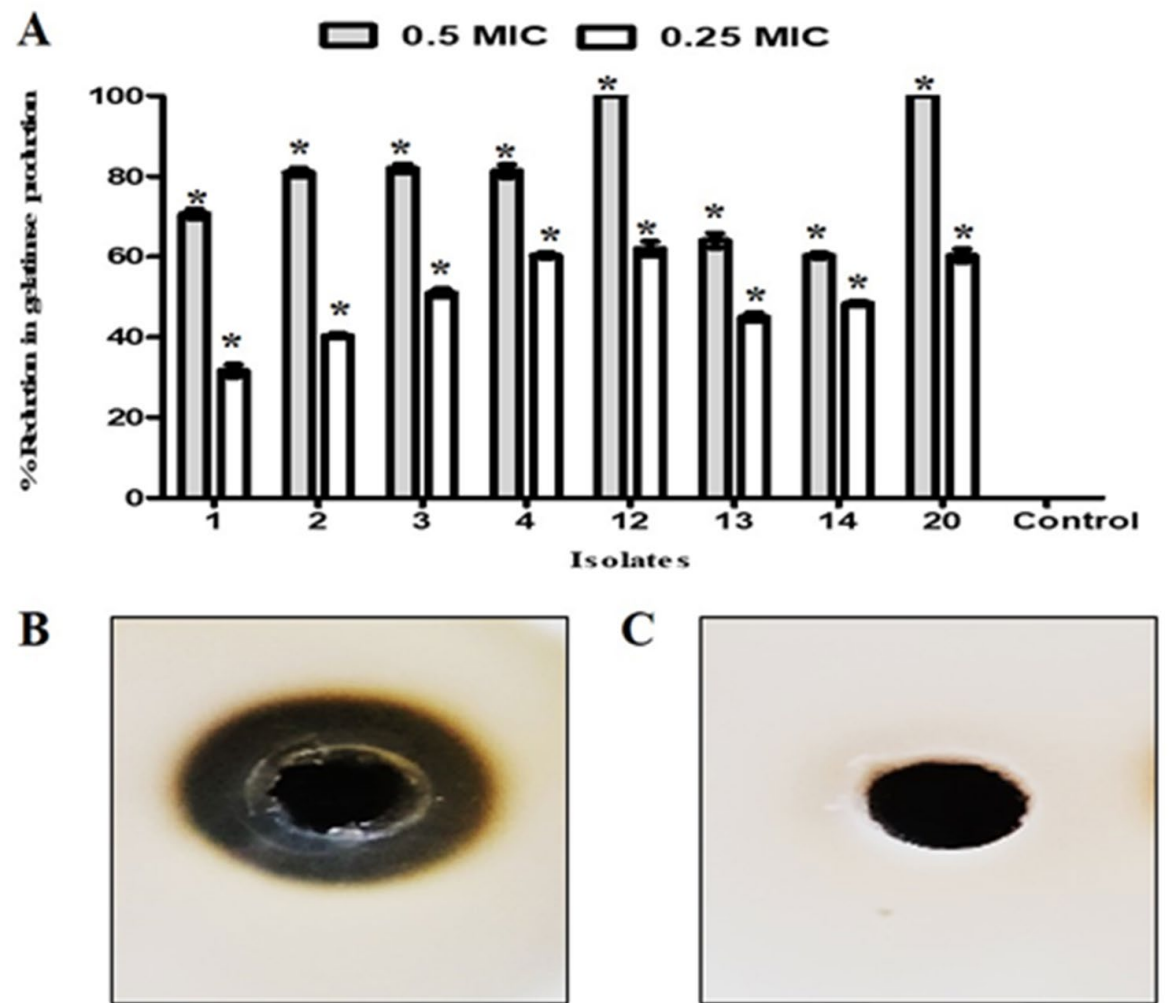
Antibiofilm activity of β-carotene against *E. faecalis* was dependent on inhibition of gelatinase enzyme action. The bar chart represents the percentages of reduction in gelatinase after treatment with 0.5 and 0.25 MIC of β-carotene (**A**). Gelatin agar plate illustrated the gelatin hydrolysis zone by the gelatinase enzyme produced by *E. faecalis* 20 (**B**). Gelatin agar plate showed the inhibition of the production of gelatinase after treatment of *E. faecalis* 20 with β-carotene (0.5 MIC) (**C**). Each bar represents the average value of triplicate measurements, and the asterisk indicates a statistically significant difference at *P* < 0.05 compared to the control

The gelatin agar plate was utilized to inspect the effect of β-carotene on gelatinase production by *E. faecalis* isolates. Our results showed that the treatment with β-carotene significantly reduced the production of gelatinase in all tested isolates compared to untreated cells (Fig. 12). The percentages of reduction were 60–100% and 32–62% after treatment with 0.5 and 0.25 MICs, respectively.

#### β-Carotene downregulated the expression of the *GelE* gene by *E. faecalis*

**Fig. 13:**
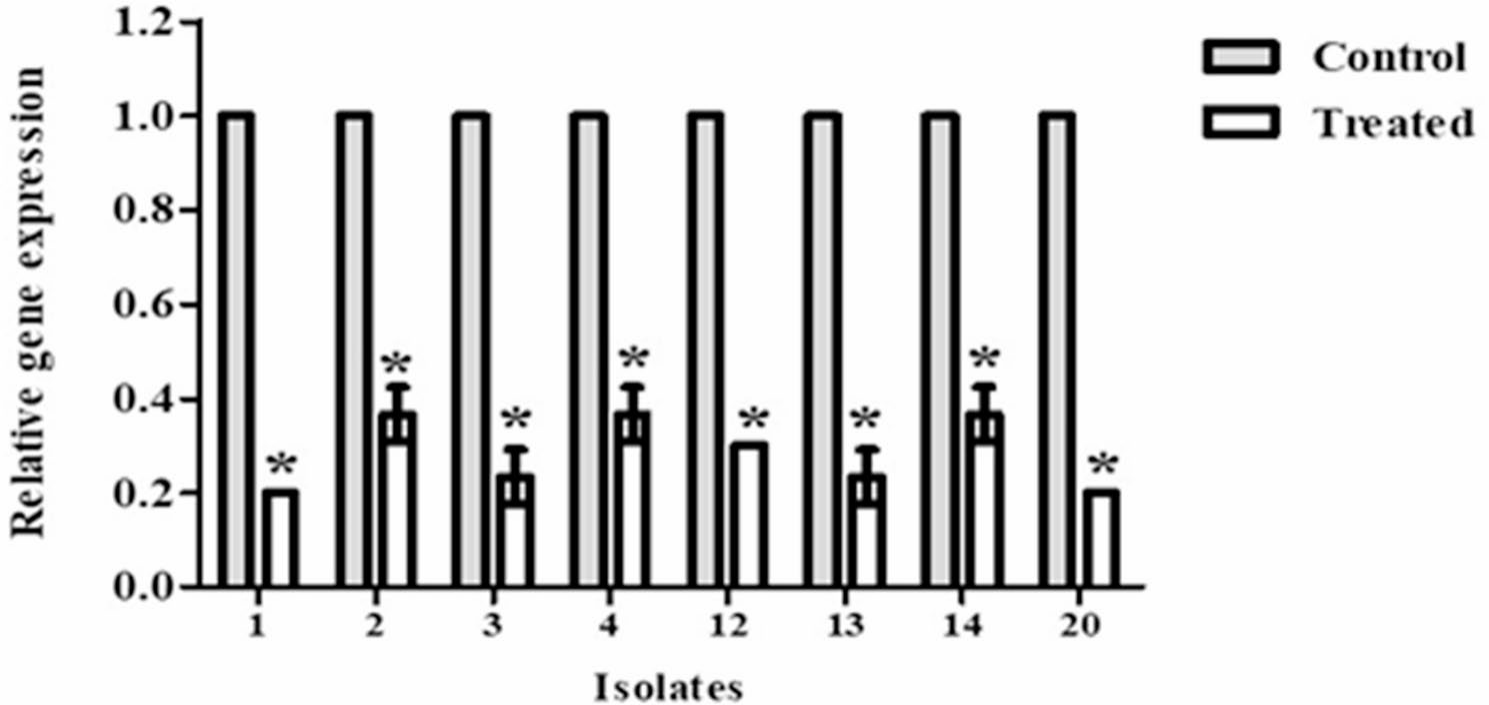
The bar chart represents the relative fold gene expression of the *gelE* gene after β-carotene treatment (0.5 MIC) compared to the control. Each bar represents the average value of triplicate measurements, and the asterisk indicates a statistically significant difference at *P* < 0.05 compared to the control

To gain insight into the molecular mechanism of β-carotene-mediated biofilm inhibition in *E. faecalis*, the expression profile of the gelatinase gene, *gelE*, was analyzed. Transcript results revealed that β-carotene significantly (*P* < 0.05) downregulated the expression of the *gelE* gene, resulting in the inhibition of biofilm formation. The percentages of reduction in the expression of the *gelE* gene after treatment with 0.5 MIC β-carotene ranged from 63 to 80% (Fig. 13).

#### Gelatinase enzyme-β-carotene complex formation inhibited the biofilm formation by *E. faecalis*

**Fig. 14:**
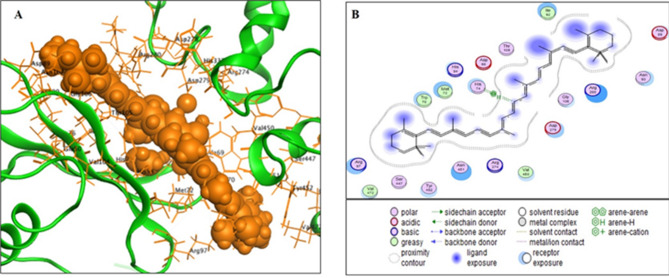
In silico analysis of the binding of gelatinase protein of *E. faecalis* to β-carotene. The three-dimensional binding mode of β-carotene with gelatinase protein (the β-carotene is colored in orange) (**A**). The two-dimensional binding mode of β-carotene with gelatinase protein (**B**)

The interaction between β-carotene and the gelatinase protein was investigated to better understand the likely mechanism by which it can produce its antibiofilm activity against *E. faecalis*. Molecular docking was carried out with the Glide ligand-docking module in standard precision mode. The findings demonstrated that β-carotene was oriented correctly in the gelatinase protein’s active region, with a substantial docking score of −9 kcal mol⁻¹. As illustrated in Fig. 14, β-carotene formed a complex with the gelatinase protein by establishing a strong hydrogen bond with the histidine (His) amino acid 74. The provided data emphasized that the antibiofilm activity of β-carotene against *E. faecalis* was linked to the inhibition of the significant role of gelatinase enzyme in biofilm formation.

## Discussion

The carotenoid pigments are produced under the control of various factors, including pH, type of medium, incubation period, temperature, and other factors [7]; therefore, these factors were adjusted in the current study for enhancing carotenoid pigment production by *M. luteus*. It was observed in our study that the TSB, shaking conditions, temperature of 37 °C, pH = 7, incubation time of 4 days, IS = 2%, sodium chloride, glycerol, and tryptone were associated with the highest productivity of carotenoid pigment. According to Rezaeeyan et al. (2017), the TSB medium was the preferred medium for carotenoid production by the *Kocuria* sp. strain QWT-12 [44], which may be due to the components of the medium that are needed for the pigment’s production [45]. Also, Mantzouridou et al. (2002) discovered that agitation increased β-carotene production because the oxygen request of a fermentation procedure was satisfied by agitating the broth [46]. In agreement with our study, Fatima et al. (2022) reported that the best temperature for the production of *Micrococcus* species’ pigment was 37 °C [47]. In addition, it was discovered that the ideal pH for carotenoid production was 7 [48]. Following our findings, Kandasamy et al. (2024) discovered that *M. luteus* produced pigment progressively from day 1 to day 4, and then after 4 days of incubation, there was a small decrease in pigment production [45]. As well, the production of carotenoid pigment by *Micrococcus* species was optimal with an IS of 2% [47]. The outcome of salinity was discussed in a previous study, which reported that sodium chloride improved carotenoid production by *Micrococcus* species [47]. Coming in agreement with our data, it was found that the addition of glycerol resulted in an elevation in carotenoid concentration by 7.5-fold in *Rhodococcus opacus* PD630 [49], and according to Deveikaite & Zvirdauskiene (2023), the addition of tryptone supported cell growth as well as pigment production [50].

This is the first report to study the effect of amino acids, organic solvents, organic acids, ferric sources, and phosphate sources on bacterial carotenoid production. It was found that tryptophan, glutaric acid, toluene, ferric sulphate, and disodium hydrogen phosphate significantly increased pigment production in *M. luteus*. According to Hajjaj et al. (2012), amino acids improved the yield of red pigments from *Monascus ruber* ATCC 96,218, and it was reported that His was the most valuable amino acid [51]. In agreement with Yahya et al. (2024), the yield of phycocyanin from microalga *Arthrospira maxima* was enhanced after the addition of 0.625 mmol glutamic acid to the medium [52]. Additionally, the creation of pyocyanin by *Pseudomonas aeruginosa* was upgraded by 275% when toluene (0.2%, v/v) was added to the medium [1]. In accordance with Peng et al. (2024), the production of fucoxanthin by *Conticribra weissflogii* was significantly affected by iron concentration [53]. Furthermore, Sanpapao et al. (2023) reported that phosphorus enhanced the production of carotenoids in *Dunaliella salina* NUAC09 [54]. Collectively, using PBD and CCD, submerged fermentation of *M. luteus* (M6) in TSB medium at an agitation speed of 120 rpm, a temperature of 32.5 °C, pH = 7, an incubation time of 96 h, an IS of 2%, and supplementation of the medium with sodium chloride (12.5 g/l), tryptophan (12.5 g/l), glutaric acid (5 g/l), toluene (12.5%), and disodium hydrogen phosphate (5 g/l) led to a 6-fold improvement in carotenoid yield.

The identification of the extracted carotenoid showed that the measured R_f_ of our extract was identical to β-carotene’s R_f_ value (R_f_: 0.89), as discussed by Gurkok & Sumeyra (2022) [55]. Moreover, the resultant DSC curve of the extracted pigment showed a sharp endothermic peak at nearly 180 °C, which was the characteristic peak of β-carotene [56]. The spectra of FTIR for our pigment and standard β-carotene nearly exhibited similar vibration bands at 3418, 2935, 2874, 1710, 1451, and 1158 cm^−1^, which confirmed that the produced pigment was β-carotene, and the resultant vibration bands were considerably the same vibration bands of β-carotene that were reported by Uzun et al. [57]. The pigment’s identity was further proven by means of HPLC, showing an identical retention time to standard one (4.9 min), which was typical of the retention time of β-carotene (4.9 min) in the study conducted by Hagaggi and Abdul-Raouf [27].

It is important to consider the stability of carotenoids during storage to get highly active ones. Therefore, we conducted a stability study, and we found that the extracted β-carotene was affected by the type of solvent and temperature. In addition, it was observed in our study that preservation of β-carotene solution in DMSO at 4 °C was optimal to achieve a 1-year stable solution. In agreement with Lu et al., who reported the rapid deterioration of carotenoids when exposed to various environmental factors, including light, high heat, acids, and solvents [58].

The purified β-carotene’s safety was examined through the cytotoxicity assay against HDFs, and it was found that the extracted β-carotene was safe at concentrations below 250 µg/ml. Generally, the toxicity of β-carotene is low [59], and it was known that β-carotene was the first bio-pigment successfully allowed as a food ingredient in 1995 [60]. Furthermore, the safety of β-carotene was investigated, and after clinical pathology and histological assessments of rats (β-carotene dose = 500 mg/kg), no negative effects were noted [61].

According to Akshaya et al. (2023), the nosocomial pathogen *E. faecalis* is essential to the pathophysiology of several illnesses, such as endocarditis, urinary tract infections, and recurrent root canal infections [62]. Thus, the antibacterial activity of the produced pigment was investigated against *E. faecalis*; it was reported for the first time that the produced β-carotene had a significant antibacterial action against MDR *E. faecalis*, with inhibition zones ranging from 13 mm to 32 mm and MICs ranging from 3.75 to 30 µg/ml. In accordance with our study, Hagaggi and Abdul-Raouf (2023) reported that the β-carotene pigment from *Citricoccus parietis* AUCs exerted a promising antibacterial activity against *S. aureus*, *Streptococcus agalactiae*, *P. aeruginosa*, and *Klebsiella pneumoniae* with variable inhibition zone diameters according to bacterial species [27].

A significant issue in hospital settings is the development of biofilms on medical devices, such as urinary catheters [62]. Many pathogens can form biofilms on these devices, including *E. faecalis*, *S. aureus*, *Proteus mirabilis*, and *P. aeruginosa*, causing catheter-associated urinary tract infections (CAUTIs) [63]. *E. faecalis* has been a major cause of CAUTIs in recent decades, and because of its resistance to heat and aseptic solutions as well as its innate and acquired resistance to several antibiotics, including vancomycin, its treatment choices are becoming more and more limited [64]. The most common cause of ineffectiveness of treatments against enterococcal CAUTIs is their ability to form biofilm on urinary catheters, which increases enterococcal antibiotic resistance [65, 66]. Consequently, the ideal approach to overcome CAUTIs is coating urinary catheters with a potent antimicrobial agent that prevents colonization of uropathogens and formation of drug-resistant biofilms [67]. Multiple urinary catheter coating agents have been described with variable effectiveness, including silver alloy, chlorhexidine, triclosan, antibiotics, antimicrobial peptides, bacteriophages, benzalkonium chloride, silver nanoparticles, polyacrylic acid, and glutaraldehyde [68, 69]. For the first time in our study, soaking the silicon catheters in β-carotene coating solution (0.5 MIC) under static conditions for 1 h interfered with biofilm formation by *E. faecalis* on urinary catheters, and the percentages of inhibition of biofilm ranged from 41.6 to 75.3%. Moreover, the antibiofilm action of the produced β-carotene was confirmed by the reduction in biofilm biomass after the investigation with the light microscope and CLSM. The antibiofilm activity of carotenoid was discussed by Çobanoğlu & Yazıcı (2022), who found that *P. aeruginosa*’s biofilm was reduced by 50% in the presence of 2–4 µg/ml of the *Rhodococcus* sp. SC1 carotenoid pigment, becoming a non-biofilm producer at a concentration of 32 µg/ml [70]. In addition, coating a silicon catheter with nitrofurazone resulted in lowering the colonization and biofilm formation by *E. faecalis*, compared to catheters without nitrofurazone coating [71]. Furthermore, our study examined the mechanistic pathway of antibiofilm activity of the produced β-carotene by phenotypic, genotypic, and molecular docking analysis. It was found that the antibiofilm action of β-carotene against *E. faecalis*’s biofilms was linked to the inhibition of the production of gelatinase enzyme. As discussed by Zheng (2018), *E. faecalis*’s gelatinase plays a crucial role in the adherence of bacteria and the development of biofilm [72].

## Conclusion

The current study provided a wide-ranging investigation of the idealistic medium compositions and fermentation parameters for carotenoid pigment production by *M. luteus* (M6 isolate). As well as this study investigated for the first time the effect of amino acids, organic solvents, organic acids, ferric, and phosphate on carotenoid production by M6 isolate. The optimal physico-chemical conditions were TSB medium, agitation speed of 120 rpm, temperature of 32.5 °C, pH = 7, incubation time of 96 h, 2% IS, sodium chloride (12.5 g/l), tryptophan (12.5 g/l), glutaric acid (5 g/l), toluene (12.5%), and disodium hydrogen phosphate (5 g/l). The stability studies showed that storage of the produced β-carotene in DMSO at 4 °C was optimal. For the first time, it was reported the antibacterial and antibiofilm activities of the purified stable β-carotene against *E. faecalis* were reported. Additionally, coating of silicon catheters by β-carotene reduced the biofilm formation by *E. faecalis* by 75.3%, and the antibiofilm action was dependent on the inhibition of gelatinase enzyme production. Future application of β-carotene-coated catheters for preventing biofilm formation will be promising.

## Supplementary Information

Supplementary Material 1

## Data Availability

No datasets were generated or analysed during the current study.
