# Supplementary material for: Coating silicon catheters with the optimized and stable carotenoid bioproduct from Micrococcus luteus inhibited the biofilm formation by multidrug-resistant Enterococcus faecalis via downregulation of GelE gene expression
Source: Microb Cell Fact. 2025 Aug 18;24:186. doi: 10.1186/s12934-025-02808-8 (PMC12359931; doi:10.1186/s12934-025-02808-8)
Supplement: Supplementary file 1 — Supplementary Material 1 [file 12934_2025_2808_MOESM1_ESM.docx]

**
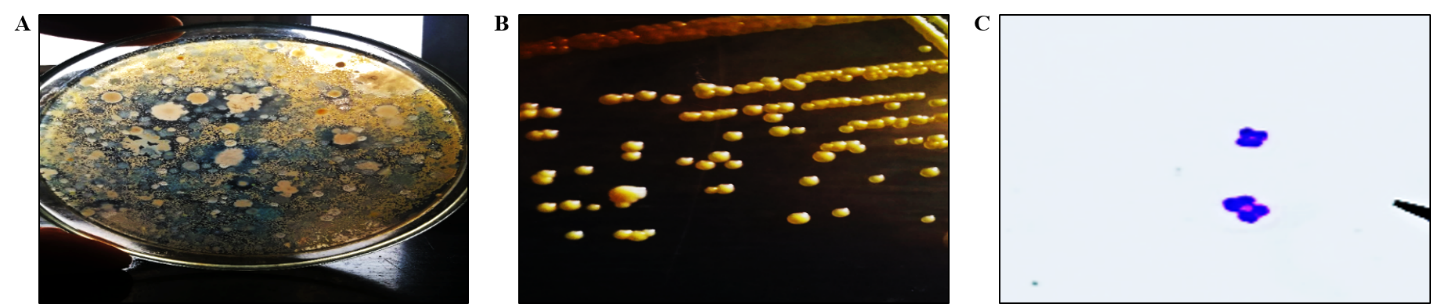
**

**Fig. S1** The yellow-pigmented colonies of *M. luteus* from the soil sample (**A**). The characteristic yellow colonies of *M. luteus* on nutrient agar (**B**). Tetrad arrangement of *M. luteus* under 100-X magnification using a light microscope (**C**).

**
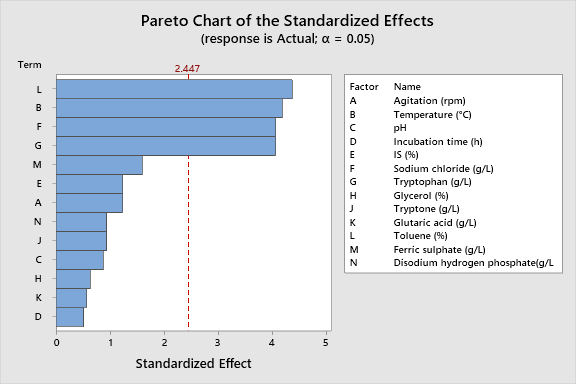
**

**Fig. S2** Pareto chart of PBD.

**
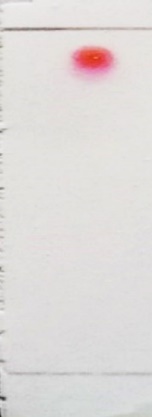
**

**Fig. S3** The TLC plate showed a retardation factor of 0.89 (characteristic of β-carotene).

**Table S1** The examined physicochemical parameters in OFAT experiments.

| **Parameters** | **Values** | | | | | | | | | |
| --- | --- | --- | --- | --- | --- | --- | --- | --- | --- | --- |
| **Medium** | Peptone broth (PB) | NB | | | Luria Bertani broth (LBB) | | Tryptic soya broth (TSB) | | | Brain heart infusion broth (BHIB) |
| **Agitation** (rpm) | 0 | 30 | | | 60 | | 120 | | | |
| **Temperature** (℃) | 25 | 30 | | | 37 | | 40 | | | |
| **pH** | 5 | 6 | | | 7 | | 8 | | | 9 |
| **Incubation time** (h) | 24 | 48 | | | 72 | | 96 | | | 120 |
| **Inoculum size** (%) | 1 | 2 | | | 3 | | 4 | | | |
| **Inorganic salt** (5 g/l) | Magnesium bromide | | Potassium bromide | | | Calcium bromide | | | Sodium chloride | |
| **Amino acid** (5 g/l) | Tryptophan | | | Serine | | | Arginine | | | |
| **Carbon source** (5 g/l) | Mannitol | Glycerol | | | Sucrose | | | Glucose | | |
| **Nitrogen source** (5 g/l) | Peptone | Tryptone | | | Ammonium sulphate | | | Sodium nitrate | | |
| **Organic acid** (5 g/l) | Glutaric acid | | | Salicylic acid | | | Ascorbic acid | | | |
| **Organic solvent** (0.5%) | Benzene | | | Toluene | | | Petroleum ether | | | |
| **Ferric** (5 g/l) | Ferric chloride | | | Ferric hydroxide | | | Ferric sulphate | | | |
| **Phosphate** (5 g/l) | Dipotassium hydrogen phosphate | | | Disodium hydrogen phosphate | | | Diammonium hydrogen phosphate | | | |

**Table S2** Sequences of primers used in qRT-PCR.

| Genes | Primer sequences | References |
| --- | --- | --- |
| *gelE* | F: 5'- TATGACAATGCTTTTTGGGAT -3'  R: 5'- AGATGCACCCGAAATAATATA -3 | [41] |
| *16s rRNA* (internal control) | F: 5'-GTCGAACGCTTCTTTCCTCC -3'  R: 5'- AGCGCCTTTCACTCTTATGC -3' | [42] |

**Table S3** Result of PBD experiment.

| **Run** | **Agitation (rpm)** | **Temperature (°C)** | **pH** | **Incubation time (h)** | **IS (%)** | **Sodium chloride (g/l)** | **Tryptophan (g/l)** | **Glycerol (%)** | **Tryptone (g/l)** | **Glutaric acid (g/l)** | **Toluene (%)** | **Ferric sulphate (g/l)** | **Disodium hydrogen phosphate (g/l)** | **Actual** | **Predicted** |
| --- | --- | --- | --- | --- | --- | --- | --- | --- | --- | --- | --- | --- | --- | --- | --- |
| 1 | 120 | 37 | 5 | 24 | 1 | 0 | 5 | 0 | 5 | 0 | 5 | 5 | 5 | 0.85 | 0.869 |
| 2 | 0 | 25 | 5 | 24 | 1 | 0 | 0 | 0 | 0 | 0 | 0 | 0 | 0 | 0.2 | 0.294 |
| **3** | **120** | **37** | **7** | **96** | **2** | **5** | **5** | **0** | **0** | **5** | **5** | **0** | **5** | **0.9** | **0.956** |
| 4 | 0 | 25 | 5 | 96 | 1 | 5 | 0 | 5 | 5 | 5 | 5 | 0 | 0 | 0.69 | 0.662 |
| 5 | 120 | 37 | 7 | 24 | 1 | 5 | 5 | 0 | 5 | 5 | 0 | 0 | 0 | 0.83 | 0.823 |
| 6 | 120 | 37 | 7 | 96 | 1 | 0 | 5 | 5 | 0 | 5 | 5 | 0 | 0 | 0.82 | 0.84 |
| 7 | 120 | 37 | 5 | 24 | 2 | 5 | 0 | 5 | 5 | 0 | 0 | 0 | 0 | 0.72 | 0.702 |
| 8 | 120 | 25 | 7 | 96 | 2 | 5 | 0 | 0 | 5 | 5 | 0 | 5 | 5 | 0.63 | 0.691 |
| 9 | 120 | 25 | 7 | 24 | 2 | 5 | 5 | 5 | 0 | 0 | 5 | 5 | 0 | 0.86 | 0.894 |
| 10 | 0 | 37 | 7 | 96 | 2 | 0 | 0 | 5 | 5 | 0 | 5 | 5 | 0 | 0.73 | 0.77 |
| 11 | 0 | 25 | 5 | 24 | 2 | 0 | 5 | 0 | 5 | 5 | 5 | 5 | 0 | 0.77 | 0.718 |
| 12 | 0 | 25 | 7 | 24 | 2 | 0 | 5 | 5 | 5 | 5 | 0 | 0 | 5 | 0.59 | 0.601 |
| 13 | 120 | 25 | 5 | 24 | 1 | 5 | 0 | 5 | 0 | 5 | 5 | 5 | 5 | 0.74 | 0.739 |
| 14 | 120 | 25 | 7 | 96 | 1 | 0 | 0 | 0 | 5 | 0 | 5 | 0 | 5 | 0.62 | 0.588 |
| 15 | 0 | 25 | 7 | 96 | 1 | 5 | 5 | 0 | 0 | 0 | 0 | 5 | 0 | 0.71 | 0.663 |
| 16 | 0 | 37 | 5 | 96 | 1 | 5 | 5 | 5 | 5 | 0 | 0 | 5 | 5 | 0.85 | 0.856 |
| 17 | 120 | 37 | 5 | 96 | 2 | 0 | 0 | 0 | 0 | 5 | 0 | 5 | 0 | 0.64 | 0.604 |
| 18 | 120 | 25 | 5 | 96 | 2 | 0 | 5 | 5 | 0 | 0 | 0 | 0 | 5 | 0.62 | 0.58 |
| 19 | 0 | 37 | 7 | 24 | 2 | 5 | 0 | 0 | 0 | 0 | 5 | 0 | 5 | 0.87 | 0.814 |
| 20 | 0 | 37 | 7 | 24 | 1 | 0 | 0 | 5 | 0 | 5 | 0 | 5 | 5 | 0.61 | 0.586 |

**Table S4** Response surface model analysis by ANOVA.

| **Source** | **DF** | **Adj SS** | **Adj MS** | **F-Value** | **P-Value** |  |
| --- | --- | --- | --- | --- | --- | --- |
| Model | 14 | 4.92267 | 0.35162 | 216.66 | 0.000 | Significant |
| Linear | 4 | 0.24165 | 0.06041 | 37.22 | 0.000 |  |
| Temperature (°C) | 1 | 0.01392 | 0.01392 | 8.58 | 0.010 |  |
| Sodium chloride (g/l) | 1 | 0.03043 | 0.03043 | 18.75 | 0.001 |  |
| **Tryptophan (g/l)** | 1 | 0.14897 | 0.14897 | 91.79 | **0.000** |  |
| **Toluene (%)** | 1 | 0.04368 | 0.04368 | 26.91 | **0.000** |  |
| Square | 4 | 4.77499 | 1.19375 | 735.57 | 0.000 |  |
| **Temperature (°C)*Temperature (°C)** | 1 | 3.11669 | 3.11669 | 1920.46 | **0.000** |  |
| **Sodium chloride (g/l)*Sodium chloride (g/l)** | 1 | 0.79833 | 0.79833 | 491.92 | **0.000** |  |
| **Tryptophan (g/l)*Tryptophan (g/l)** | 1 | 0.28158 | 0.28158 | 173.50 | **0.000** |  |
| **Toluene (%)*Toluene (%)** | 1 | 0.65682 | 0.65682 | 404.72 | **0.000** |  |
| 2-Way Interaction | 6 | 0.01325 | 0.00221 | 1.36 | 0.289 |  |
| Temperature (°C)*Sodium chloride (g/l) | 1 | 0.01000 | 0.01000 | 6.16 | 0.025 |  |
| Temperature (°C)*Tryptophan (g/l) | 1 | 0.00000 | 0.00000 | 0.00 | 1.000 |  |
| Temperature (°C)*Toluene (%) | 1 | 0.00202 | 0.00202 | 1.25 | 0.280 |  |
| Sodium chloride (g/l)*Tryptophan (g/l) | 1 | 0.00022 | 0.00022 | 0.14 | 0.715 |  |
| Sodium chloride (g/l)*Toluene (%) | 1 | 0.00090 | 0.00090 | 0.55 | 0.467 |  |
| Tryptophan (g/l)*Toluene (%) | 1 | 0.00010 | 0.00010 | 0.06 | 0.807 |  |
| Error | 16 | 0.02597 | 0.00162 |  |  |  |
| Lack-of-Fit | 10 | 0.02548 | 0.00255 |  |  |  |
| Pure Error | 6 | 0.00049 | 0.00008 |  |  |  |
| Total | 30 | 4.94864 |  |  |  |  |

**Table S5** VITEK 2 compact system biochemical profile of *E. faecalis*.

| Well number | Test | *E. faecails* | Well number | Test | *E. faecails* |
| --- | --- | --- | --- | --- | --- |
| 2 | **AMY** | + | **32** | **POLYB** | + |
| 4 | **PIPLC** | - | **37** | **dGAL** | **+** |
| 5 | **Dxyl** | - | **38** | **dRIB** | **+** |
| 8 | **ADHI** | + | **39** | **ILATk** | - |
| 9 | **BGAL** | - | **42** | **LAC** | **+** |
| 11 | **AGLU** | + | **44** | **NAG** | **+** |
| 13 | **APPA** | - | **45** | **dMAL** | **+** |
| 14 | **CDEX** | + | **46** | **BACI** | **+** |
| 15 | **AspA** | + | **47** | **NOVO** | **+** |
| 16 | **BGAR** | - | **50** | **NC6.S** | **+** |
| 17 | **AMAN** | - | **52** | **dMAN** | **+** |
| 19 | **PHOS** | - | **53** | **dMNE** | **+** |
| 20 | **LeuA** | - | **54** | **MBdG** | **+** |
| 23 | **ProA** | - | **56** | **PUL** | - |
| 24 | **BGURr** | - | **57** | **dRAF** | - |
| 25 | **AGAL** | - | **58** | **0129R** | - |
| 26 | **PyrA** | + | **59** | **SAL** | **+** |
| 27 | **BGUR** | - | **60** | **SAC** | **+** |
| 28 | **AlaA** | + | **62** | **dTRE** | **+** |
| 29 | **TyrA** | + | **63** | **ADH2s** | **+** |
| 30 | **Dsor** | + | **64** | **OPTO** | **+** |
| 31 | **URE** | - |  |  |  |

**Table S6** Resistance pattern of *E. faecalis* (n = 20)

| **Resistotypes** | **Resistance pattern** | **Isolates** | **Percentage** |
| --- | --- | --- | --- |
| **I** | AP, CIP, E, TE | 1, 10, 15, 19 | 20% |
| **II** | CIP, E, TE | 2, 3, 5, 6, 7, 8, 9, 11, 12, 13, 14, 15, 16, 17, 18, 19, 20. | 75% |
| **III** | AP, E, TE | 4 | 5% |

**AP**: ampicillin, **CIP**: ciprofloxacin, **E**: erythromycin, **TE**: tetracycline, **VAN**: vancomycin.

**Table S7** Average ODs of *E. faecalis* (n = 20) at 595 nm.

| Isolate | Average OD at 595 nm | Biofilm | Isolate | Average OD at 595 nm | Biofilm |
| --- | --- | --- | --- | --- | --- |
| 1 | 0.982 | Strong | **11** | 0.351 | Moderate |
| 2 | 0.834 | Strong | **12** | 0.826 | Strong |
| 3 | 0.798 | Strong | **13** | 1.32 | Strong |
| 4 | 0.956 | Strong | **14** | 0.842 | Strong |
| 5 | 0.32 | Moderate | **15** | 0.304 | Moderate |
| 6 | 0.212 | Moderate | **16** | 0.311 | Moderate |
| 7 | 0.367 | Moderate | **17** | 0.325 | Moderate |
| 8 | 0.34 | Moderate | **18** | 0.274 | Moderate |
| 9 | 0.283 | Moderate | **19** | 0.21 | Moderate |
| 10 | 0.193 | Weak | **20** | 0.924 | Strong |
